# Perceptions, attitudes, and curriculum reflections: exploring healthcare students’ engagement with basic medical sciences in Saudi Arabia

**DOI:** 10.3389/fmed.2026.1791516

**Published:** 2026-03-31

**Authors:** Khalid Al-Hamad, Abdullah Aljuhani, Turki Almutairi, Hanan Shanab, Reem Almutairi, Angel Joseph, Ilango Saraswathi, Jayakumar Saikarthik

**Affiliations:** 1 Department of Oral and Maxillofacial Diagnostic Sciences, College of Dentistry, Qassim University, Buraydah, Saudi Arabia; 2 College of Dentistry, Majmaah University, Al Majma'ah, Saudi Arabia; 3 Department of Maxillofacial Surgery and Diagnostic Sciences, College of Dentistry, Majmaah University, Al Majma’ah, Saudi Arabia; 4 Department of Restorative Dentistry and Prosthodontics, College of Dentistry, Majmaah University, Al Majma’ah, Saudi Arabia; 5 Mauli Medical College and Research Institute, Buldhana, India; 6 Department of Business Administration, Manipal Academy of Higher Education, Manipal, India

**Keywords:** attitude, basic medical science, curriculum, healthcare students, perception, Saudi Arabia

## Abstract

**Background:**

Understanding healthcare students’ perceptions of basic medical sciences (BMS) and curriculum structure is essential for informed educational reform in Saudi Arabia. This study explored attitudes toward BMS subjects and curriculum design among undergraduate healthcare students across medicine, dentistry, nursing, and applied medical sciences programs.

**Methods:**

A descriptive, cross-sectional study was conducted from June to December 2023 among 397 students from public and private universities in and around Riyadh, Saudi Arabia. Data were collected via a validated, self-administered online questionnaire using snowball sampling. The instrument assessed interest in BMS subjects and careers, attitudes toward foundational sciences, and curriculum structure preferences. Exploratory factor analysis with varimax rotation, Mann–Whitney *U*, Kruskal–Wallis, multiple regression, and Spearman correlation were performed using SPSS v29 (IBM Corp.); *p* < 0.05 was considered statistically significant.

**Results:**

Dentistry students comprised 45.1% of respondents. While 56.7% showed strong interest in BMS courses, only 26.2% intended to pursue BMS careers due to perceived lack of excitement (14.4%) and financial incentives (10.6%). anatomy (80.9%), pathology (79.1%), and physiology (68.0%) were rated as the most clinically relevant; biochemistry was rated the lowest (27.7%). A majority (70.3%) supported integrating BMS into clinical teaching and preferred integrated, problem-based learning (PBL) curricula. PBL students expressed significantly more positive BMS attitudes (*p* < 0.05). Sex, academic program, and university type influenced attitudes. Positive BMS perceptions correlated moderately with PBL support (*r* = 0.366, *p* < 0.001).

**Conclusion:**

Students value clinically relevant BMS but show limited career interest. PBL and integrated curricula enhance engagement. Targeted mentorship may strengthen BMS career pathways.

## Introduction

Basic medical sciences (BMS), encompassing disciplines such as anatomy, physiology, biochemistry, pathology, pharmacology, and microbiology, serve as the foundational pillars of healthcare education. These subjects provide essential knowledge required for understanding disease processes, interpreting diagnostic tests, and applying therapeutic strategies in clinical practice (1). The acquisition of BMS knowledge is not only crucial during the pre-clinical years but also significantly influences clinical reasoning and decision-making during later stages of training and professional practice (2, 3).

Despite their fundamental role, healthcare students’ attitudes and perceptions toward BMS can vary widely. In many settings, students report difficulties in understanding and retaining BMS content due to its abstract nature, high volume, and sometimes limited immediate clinical relevance (4). Moreover, the compartmentalized teaching of BMS subjects in traditional curricula often leads to fragmented understanding, further impacting students’ engagement and motivation (5). Consequently, medical educators have increasingly emphasized curriculum reform, favoring integrated curricula that include problem-based learning (PBL) models, which blend basic sciences with clinical scenarios to foster contextual understanding and improve long-term retention (6).

A conventional curriculum refers to a discipline-based model delivering BMS as discrete, sequentially organized courses, whereas an integrated curriculum organizes content through horizontal and vertical integration across disciplines and academic years (7, 8). Problem-based learning (PBL) is a student-centered pedagogical strategy that may be embedded within either model, though it is most commonly implemented within integrated frameworks (9). In Saudi Arabia, healthcare programs across medicine, dentistry, nursing, and applied medical sciences exhibit considerable curricular heterogeneity, ranging from conventional discipline-based models to PBL-integrated curricula. The structural embedding of BMS varies considerably across disciplines: medical and dental curricula generally allocate 2–3 years of dedicated BMS instruction, with some institutions adopting PBL-integrated models, whereas nursing and applied medical sciences programs deliver BMS in a more condensed format emphasizing applied clinical competencies over in-depth foundational coverage (10–12).

Saudi dental programs (7 years, including a preparatory year) allocate the first 2 years predominantly to BMS subjects—including anatomy, physiology, biochemistry, pathology, and microbiology—followed by a preclinical simulation phase before clinical training commences in the fourth year (13, 14). This extended BMS phase is comparable in duration and depth to that of medical programs. In contrast, nursing programs (4 + 1 years of internship) deliver BMS content within the first two semesters as prerequisite courses, after which students transition into specialized nursing modules (15) with limited BMS revisiting. Applied medical sciences programs (4–5 years, depending on the discipline) follow a similar condensed pattern, with BMS concentrated in the first 2 years and subsequent emphasis on discipline-specific technical competencies such as laboratory sciences, radiological technology, or respiratory therapy (16). This inter-program variation in BMS exposure mirrors international findings where nursing and allied health students perceive BMS as less clinically relevant than their medical and dental counterparts (17, 18). It is also important to acknowledge that there is a severe dearth of literature assessing the type of curriculum within Saudi Arabia, especially in the case of nursing and applied medical sciences programs.

In a global perspective, medical curricula in the United Kingdom and the Netherlands have largely transitioned to fully integrated models with early clinical immersion (8, 19, 20), while programs in South Asia and parts of Africa frequently retain conventional discipline-based structures (21–23). In dental education, a similar tension exists: while the United Kingdom and Turkey employ problem-based learning approaches that integrate basic and clinical sciences and expose students to clinical cases at an early stage, countries such as Pakistan continue to use discipline-based learning with BMS concentrated in the early years (24). In nursing education, the “bioscience problem,” the persistent difficulty nursing students experience in learning and applying human biosciences, has been documented internationally for over two decades, with bioscience courses typically concentrated in the first year and taught as a largely separate discipline from clinical nursing content (25, 26). In allied/ applied health sciences, BMS is similarly positioned within a foundational preparatory phase before discipline-specific competencies, with quality assurance frameworks emphasizing the need for curriculum evaluation systems that ensure effective integration of BMS into professional practice (27). This clearly reflects heterogeneity in terms of the type of curriculum implemented across medicine, dentistry, nursing, and applied medical sciences programs both globally and within the Saudi Arabian context.

In recent years, the discourse around the teaching and learning of BMS has expanded, recognizing that students’ perceptions and attitudes are shaped not only by the content itself but also by how it is delivered. Effective pedagogical approaches, such as interactive lectures, case-based discussions, simulation, and early clinical exposure, are believed to enhance student interest and positively influence their attitude toward foundational sciences (28–30). A favorable attitude toward BMS has also been associated with improved academic performance, deeper learning, and increased confidence during clinical rotations (31, 32). Conversely, negative perceptions can lead to reduced engagement, surface learning, and challenges in applying basic science concepts in clinical contexts (30).

In Saudi Arabia, healthcare education has undergone significant transformation over the past two decades, marked by an increased focus on international accreditation standards, competency-based education, and curriculum modernization. This modernization is guided by the National Qualifications Framework for Saudi Arabia (NQF-KSA), developed by the Education and Training Evaluation Commission (ETEC), which classifies qualifications across eight hierarchical levels based on progressive learning outcomes in three domains: knowledge, skills, and values. The framework provides generic competency descriptors for each level while affording individual institutions flexibility in curricular design and delivery (33). Within healthcare programs, this institutional autonomy means that while all programs must meet the same competency-based outcome standards, the sequencing, depth, and integration of BMS content vary across institutions, partly explaining the inter-institutional variability observed in curricular models within Saudi Arabia. However, despite these structural reforms, research evaluating their effectiveness from the students’ perspective remains limited. Notably, only one prior study in Saudi Arabia has specifically investigated healthcare students’ attitudes toward BMS, revealing limited interest and identifying barriers such as a preference for clinical subjects and a lack of hands-on learning opportunities (34). Building on this, it becomes essential to recognize that Saudi students’ experiences are shaped by a set of contextual factors that may not mirror those in Western settings (35, 36). At the institutional level, the rapid growth of medical colleges and the pressure to meet accreditation standards have often resulted in curricular designs that emphasize compliance and structure over innovation in teaching (37). From a cultural perspective, clinical disciplines tend to be regarded as more prestigious and closely tied to professional identity, which can naturally shift students’ attention away from the foundational sciences (34). On the pedagogical front, while many schools have embraced integrated models, traditional lecture-based teaching and limited opportunities for active learning or early clinical exposure continue to shape how students engage with BMS (38). Taken together, these factors suggest that Saudi students’ perceptions of BMS are influenced by a blend of institutional priorities, cultural expectations, and educational practices that warrant closer exploration. International studies have highlighted variation in attitudes toward BMS across disciplines like medicine, dentistry, and nursing (18, 39–42). For instance, medical students in several contexts reported perceiving BMS as abstract and content-heavy (41, 43), while dental students often valued BMS more when taught in relation to clinical practice (18). Nursing students, on the other hand, frequently expressed difficulties in appreciating the immediate clinical relevance of BMS, which affected their motivation (42, 44). These differences point to how professional identity and curriculum design shape attitudes toward basic medical sciences. In contrast, the only Saudi study to date, Althubaiti and Althubaiti (34), did not compare across disciplines but instead emphasized barriers such as students’ preference for clinical subjects and the lack of hands-on learning opportunities. Thus, there is a notable lack of data that explore how variables such as curriculum structure, teaching methodology, and university type (public vs. private) affect students’ views toward BMS. Such data are essential for informing educational policy, designing more student-centered curricula, and enhancing the teaching and learning of BMS in line with national healthcare goals.

The present study is underpinned by expectancy–value theory (EVT) of achievement motivation (45, 46), which posits that academic and career-related decisions are governed by two core constructs: individuals’ expectations of success in a given domain and the subjective value ascribed to it, encompassing intrinsic value, utility value, attainment value, and perceived cost. This theoretical lens was selected for three reasons: first, it provides an empirically validated framework for explaining why students differentially value and engage with specific academic domains despite similar achievement levels; second, its model incorporates sociocultural influences and socializer effects as antecedents of value appraisals, allowing contextual factors such as program structure to be theoretically accommodated; and third, it was originally formulated to explain sex-based differences in achievement motivation and domain-specific academic choices (45), rendering it directly applicable to the demographic subgroup analyses undertaken in this study. Thus, this theory offers a validated framework to explain how students’ expectations of success, subjective task value, and perceived costs may influence their interest in BMS and their intention to pursue BMS-related careers, while also accounting for the influence of educational context, curriculum design, and sociocultural factors on these value judgments. Accordingly, EVT guided the study design by informing the selection of survey domains assessing perceived utility (clinical relevance ratings), intrinsic value (interest in BMS courses), attainment value (career intentions), and perceived costs (barriers to BMS careers), while also providing the interpretive lens through which findings are analyzed in the Discussion. The present study seeks to fill this gap by examining the attitudes, perceptions, and interests of undergraduate healthcare students toward basic medical sciences in universities located in and around Riyadh. Additionally, it explores students’ views on curriculum integration, instructional strategies, and the role of BMS in their academic and future professional development. By identifying key factors that influence student engagement with foundational sciences, the study aims to offer evidence-based insights to support ongoing curricular reforms and enhance the quality of healthcare education in Saudi Arabia.

## Methodology

### Study design and setting

This descriptive, cross-sectional study was conducted among undergraduate healthcare students from various public and private universities enrolled in medicine, dentistry, applied medical science, nursing, and other healthcare-related courses in and around Riyadh, Saudi Arabia. The study was conducted between June 2023 and December 2023.

Undergraduate healthcare students (including interns) enrolled in medicine, dentistry, nursing, applied medical sciences, or other healthcare-related programs (like pharmacy, audiology, and speech–language pathology, public health, etc.) at universities in and around Riyadh, Saudi Arabia, across all academic years including interns, who provided electronic informed consent were eligible. To ensure data quality, incomplete responses and those with extreme completion times—below 5 min or exceeding 30 min—indicating careless responding or survey abandonment were excluded, as response time is a validated indicator for detecting insufficient effort responding in online surveys (47, 48). Duplicate submissions were restricted via Google Forms settings. The study was conducted following the STROBE guidelines for cross-sectional studies (Supplementary Figure S1) (49).

### Sampling technique

A non-probability snowball sampling technique was used due to constraints in reaching the participants across varied locations. This technique utilizes the existing professional and social networks for the efficient recruitment of participants (50, 51).

### Sample size estimation

The sample size for this study was calculated based on previous studies using Raosoft^1^ (52, 53). The targeted programs are the undergraduate healthcare students (including interns) enrolled in medicine, dentistry, applied medical sciences, nursing, and others (pharmacy, public health, etc.) in universities located in Riyadh province. A recent study by Abdulaziz et al. mentioned that the estimated number of healthcare students enrolled in King Saud University, Riyadh, is around 20,000 (54). However, the total number of students across the entire Riyadh was unknown. Hence, the sample size was calculated based on the World Health Organization (WHO) guidance for the smallest sample size required for a prevalence study (55, 56) where the population is unknown. The sample size for this cross-sectional survey was calculated using Cochran’s formula. Assuming a 95% confidence level (*Z* = 1.96), a 5% margin of error (*e* = 0.05), and an anticipated response proportion of 50% (*p* = 0.5) due to the absence of prior data on the topic, the minimum required sample size was determined to be 384 participants. This estimate ensures sufficient statistical power and accounts for maximum variability in responses. To further accommodate potential incomplete responses or dropouts, a slightly higher number of participants were targeted. A total of 420 responses were received, out of which 8 responses were eliminated as the time taken to fill the questionnaire was extreme (more than 30 min or less than 5 min), and 15 were eliminated for being incomplete. Ultimately, a total of 397 healthcare students were included in the study, slightly exceeding the minimum requirement and strengthening the reliability of the results (response rate 94.5%).

### Survey instrument development and structure

The survey instrument was a structured, self-administered online questionnaire developed after reviewing relevant literature and validated scales to ensure content validity (41, 57). The instrument was developed in both English and Arabic.

The survey instrument comprised four sections with a total of 25 questions (sections 2, 3, and 4): Section 1 included basic details that captured demographic and academic background, including age, sex, year of study, program enrolled, curriculum type (conventional, problem-based, or other), and university type (public/private). Section 2 included a set of 8 questions regarding the students’ perception and interest in BMS, adapted from Teshome et al. (41). Section 3 included questions regarding the students’ attitudes toward BMS. It contained questions adapted from the validated tool by West et al. (57) and measured attitudes toward BMS using a five-point Likert scale (1 = highly disagree to 5 = highly agree) (minimum score of 9 and maximum score of 45) (57). Statements addressed motivation, difficulty, enjoyment, and perceived usefulness of BMS subjects. It should be noted that one item in this section (Q3: ‘Psychological factors are just as important as physical ones in the healing process’) was retained from the original validated scale but reflects a broader biopsychosocial perspective on patient care rather than attitudes specifically toward BMS content or instruction. Findings related to this item are therefore interpreted as exploratory observations. Section 4 included self-made questions regarding the students’ perception of curriculum structure. This section evaluated students’ views on the design, integration, and delivery of their academic curriculum, including opinions on subject connectivity, teaching methods, and long-term retention of knowledge. Statements are graded using a five-point Likert scale: 1 (strongly disagree), 2 (disagree), 3 (neither agree nor disagree), 4 (agree), and 5 (strongly agree). According to the Likert scale, points are allocated to each answer, and the scores of domains are computed based on their sum (Supplementary File 2) (minimum score of 6 and maximum score of 30). The instrument structure was theoretically aligned with EVT. Section 2 items captured intrinsic value (interest in BMS), utility value (clinical relevance perceptions), and cost perceptions (career barriers such as financial concerns and lack of excitement); Section 3 assessed attitudes reflecting expectancy beliefs about success and engagement with BMS; and Section 4 evaluated curriculum preferences, operationalizing how educational context shapes value judgments.

### Pilot testing

The questionnaire was pre-tested with a group of 30 students from different disciplines to ensure clarity, relevance, and time efficiency, and to establish face and content validity (58). Additionally, the content and face validity were established through expert review (59, 60) by a panel of five subject matter experts (one medical education specialist, one biostatistician, and three senior basic medical sciences faculty members). Given that the study included responses from students across diverse healthcare universities in the Riyadh region, the questionnaire underwent forward and back translation by a bilingual expert proficient in both English and Arabic to ensure linguistic equivalence. The expert panel, comprising five faculty members with extensive experience in Saudi healthcare education, subsequently assessed each item for cultural appropriateness, contextual relevance, and semantic clarity to ensure suitability for the target population. Feedback was incorporated into the final version. The internal consistency of the instrument was assessed using Cronbach’s alpha, with an overall reliability coefficient of 0.67. This value is considered to be acceptable for exploratory studies and for instruments that assess broad, multi-dimensional constructs (61).

### Exploratory factor analysis for identifying underlying dimensions of students’ attitudes

Exploratory factor analysis (EFA) was conducted to identify latent variables and to establish construct validity for the nine-item questionnaire assessing students’ perceptions toward basic medical sciences and the six-item questionnaire evaluating students’ perceptions of their curriculum. Principal component analysis with varimax rotation was applied. Factor retention was determined using the scree plot, Kaiser criterion (eigenvalue >1), and cross-validated through parallel analysis. Two factors emerged in the nine-item questionnaire: Negative attitude toward BMS and positive attitude toward BMS (Supplementary Table S1). Items 1 and 2 were included in the “negative attitude” factor, while items 3, 4, 5, 6, and 9 loaded onto the “positive attitude” factor. Items 7 and 8 did not load meaningfully onto either factor and showed weak shared variance with the underlying factors, indicating that they did not contribute significantly to the factor structure (Supplementary Table S2). Therefore, these two items were excluded from subsequent regression analysis to maintain the validity and internal consistency of the factor-based composite scores (revised minimum score of 7 and maximum score of 35). The scores for positive and negative attitudes toward BMS were calculated as the mean of the items loading on each factor, and these composite scores were used as dependent variables in the regression analysis.

Similarly, two distinct factors were identified in the six-item questionnaire: attitude toward conventional curriculum and attitude toward PBL-integrated curriculum (Supplementary Table S3). Items 1 and 3 loaded onto the “Conventional curriculum” factor, whereas items 2, 4, 5, and 6 loaded onto the “Integrated curriculum” factor. Scores for each factor were calculated by averaging the individual item scores. The exploratory factor analysis was conducted to empirically confirm the clustering of items, ensure internal consistency, and provide a validated factor structure that served as the basis for subsequent statistical analyses and interpretation of students’ perceptions of conventional versus PBL-integrated curricula.

### Data collection procedure

The questionnaire was hosted on Google Forms and distributed electronically. Duplicate responses were prevented by restricting submissions to one per respondent, and only fully completed responses were included in the final analysis. Initially, the questionnaire was shared with the healthcare students of Majmaah University through the department heads of various colleges. The students were further encouraged to share the link with their friends who were enrolled in other healthcare colleges across the universities in Riyadh. The authors also contacted faculty members across various universities in the Riyadh province and shared the questionnaire link, which was then disseminated to students via institutional channels and social media platforms such as WhatsApp and X (62).

### Data analysis

Quantitative data were analyzed using SPSS software (version 29). Parallel analysis was performed using scripts from O’Connor (63). Descriptive statistics such as frequencies, percentages, means, and medians were used to summarize the data. Mann–Whitney *U* and Kruskal–Wallis *H* tests were applied to compare responses across subgroups (e.g., sex, curriculum type, academic program, and university type). To study the association between the sociodemographic variables and students’ attitudes toward BMS and their curricula, regression analysis was performed. Multiple regression analysis was conducted using the two factors obtained from each questionnaire as the dependent variable and the sociodemographic factors recorded, such as age group, sex, year of study, academic program, type of university, and curriculum type, as independent variables to study the determinants of students’ attitudes and perceptions toward BMS and their curriculum. Spearman correlation was performed to study the correlation between students’ attitudes toward BMS and their curricula. Statistical significance was set at *p* < 0.05.

### Ethical considerations

Ethical approval was obtained from the Institutional Review Board (IRB) of Majmaah University (MUREC) (HA-01-R-088). All participants provided informed consent electronically before starting the survey. The questionnaire was anonymous, and no identifying data were collected, ensuring participant confidentiality and voluntary participation. The study was conducted in compliance with the Declaration of Helsinki.

## Results

The demographic and academic characteristics of the participants are summarized in Table 1. A total of 397 healthcare students took part in the study, with a fairly even sex-based distribution (52.1% male and 47.9% female). Most participants were between the ages of 21 and 23 years (48.1%). Students from various programs were represented, with dentistry students forming the largest group (45.1%), followed by medicine (25.4%), nursing (12.1%), applied medical sciences (10.3%), and other healthcare-related courses (7.1%). Participants spanned all academic years, including interns, with nearly equal representation from first and second years (each 21.7%). The majority were enrolled in public universities (89.2%), and more than half (56.7%) followed a conventional curriculum, while 35.5% were part of a problem-based curriculum. Seven point 8 % of respondents reported following a curriculum that included hybrid or modular models that did not fit neatly into the conventional or PBL-integrated categories and were grouped under ‘other curricula’. Academic performance was self-reported through GPA ranges across different years, with the highest proportion of students reporting a GPA between 4 and 5, especially during the preparatory and early academic years.

**Table 1:** Socio-demographic and academic characteristics of the respondents.

Variables	Subgroup	Frequency	Percentage
Age	18–20	122	30.7
	21–23	191	48.1
	24–27	76	19.1
	27–30	8	2.0
Gender	Female	190	47.9
	Male	207	52.1
Program enrolled	Medicine	101	25.4
	Dentistry	179	45.1
	Applied medical science	41	10.3
	Nursing	48	12.1
	Other healthcare related courses	28	7.1
Year of study	1st year	86	21.7
	2nd year	86	21.7
	3rd year	72	18.1
	4th year	59	14.9
	5th year	45	11.3
	Internship	49	12.3
Type of university	Private	43	10.8
	Public	354	89.2
Type of curriculum followed	Conventional curriculum – (Individual course based)	225	56.7
	Problem based curriculum	141	35.5
	Others	31	7.8

Academic performance								
Year	2–3 GPA		3–4 GPA		4–5 GPA		Not applicable	
	Frequency	Percentage	Frequency	Percentage	Frequency	Percentage	Frequency	Percentage
Preparatory year	10	2.5	26	6.5	361	90.9	–	–
1st year	11	2.8	31	7.8	296	74.6	59	14.9
2nd year	5	1.3	38	9.6	219	55.2	135	34.0
3rd year	3	0.8	22	5.5	179	45.1	193	48.6
4th year	2	0.5	10	2.5	125	31.5	260	65.5
5th year	1	0.3	4	1.0	76	19.1	316	79.6

### Perception and interest of students toward basic medical science subjects

Table 2 shows the perception and interest of students toward basic medical science subjects. The responses from 397 participants revealed a generally positive inclination toward basic medical sciences (BMS) courses and related careers. A majority of students (56.7%) expressed strong interest in BMS courses, while 36.8% reported minimal interest and only 6.5% showed no interest. However, when asked about pursuing a career in BMS, only 26.2% affirmed their intent, while a nearly equal proportion of students responded “maybe” (36.8%) or “no” (37.0%), suggesting uncertainty or hesitation among many respondents regarding long-term career commitment to the field. When it came to guiding juniors toward BMS careers, only 19.4% stated they would encourage it, whereas 23.4% would not, and 33.5% responded with “maybe.” Notably, 23.7% were unsure, indicating a need for more awareness and mentorship in the field.

**Table 2:** Perception and interest of students toward basic medical science subjects.

S. no	Question	Response	Frequency (*n* = 397)	Percentage
1	Are you interested in BMS courses?	Nil	26	6.5
		Very much	225	56.7
		Minimal	146	36.8
2	Do you plan a career in BMS?	Maybe	146	36.8
		No	147	37.0
		Yes	104	26.2
3	Will you guide your junior to join a career in BMS?	I do not know	94	23.7
		Maybe	133	33.5
		No	93	23.4
		Yes	77	19.4
4	What is the reason to not join in BMS as a career?	Family pressure	10	2.5
		Less chance of promotion	16	4.0
		Less financial growth	42	10.6
		Less thrilling field	57	14.4
		No role model	8	2.0
		None of the above	70	17.6
		Not interested	103	25.9
		Others	6	1.5
		Would like to take a career in Basic Medical Sciences	85	21.4
5	Should your BMS teachers encourage you to join this field?	No	126	31.7
		Yes	271	68.3
6	Should be number of teaching hours increase in BMS courses?	No	286	72.0
		Yes	111	28.0
7	Will an integrated curriculum increase your interest to BMS?	I do not know	175	44.1
		No	49	12.3
		Yes	173	43.6
8	Will preclinical/animal research opportunities in BMS interest you?	I do not know	113	28.5
		No	59	14.9
		Yes	225	56.7

The reasons cited for not choosing a BMS career varied. The most common reason was lack of interest (25.9%), followed by the perception that it is a “less thrilling field” (14.4%) and offers “less financial growth” (10.6%). Encouragingly, 21.4% indicated a desire to pursue a BMS career despite these concerns. Other reasons, such as family pressure (2.5%), lack of role models (2.0%), and limited promotion opportunities (4.0%), were less frequently cited.

A strong majority (68.3%) believed that BMS teachers should actively encourage students to join the field, suggesting that mentorship could play a crucial role in career decision-making. Interestingly, 72.0% of respondents felt that the current number of teaching hours in BMS was adequate, while 28.0% advocated for increased hours. Regarding curriculum design, responses were mixed—43.6% believed n PBL-integrated curriculum could enhance interest in BMS, while 12.3% disagreed, and 44.1% were unsure. Lastly, a significant proportion of students (56.7%) expressed interest in preclinical or animal research opportunities, indicating that hands-on research exposure may serve as a motivational factor to pursue a BMS-related career.

### Clinical relevance rating of the basic medical science disciplines

**Figure 1:**
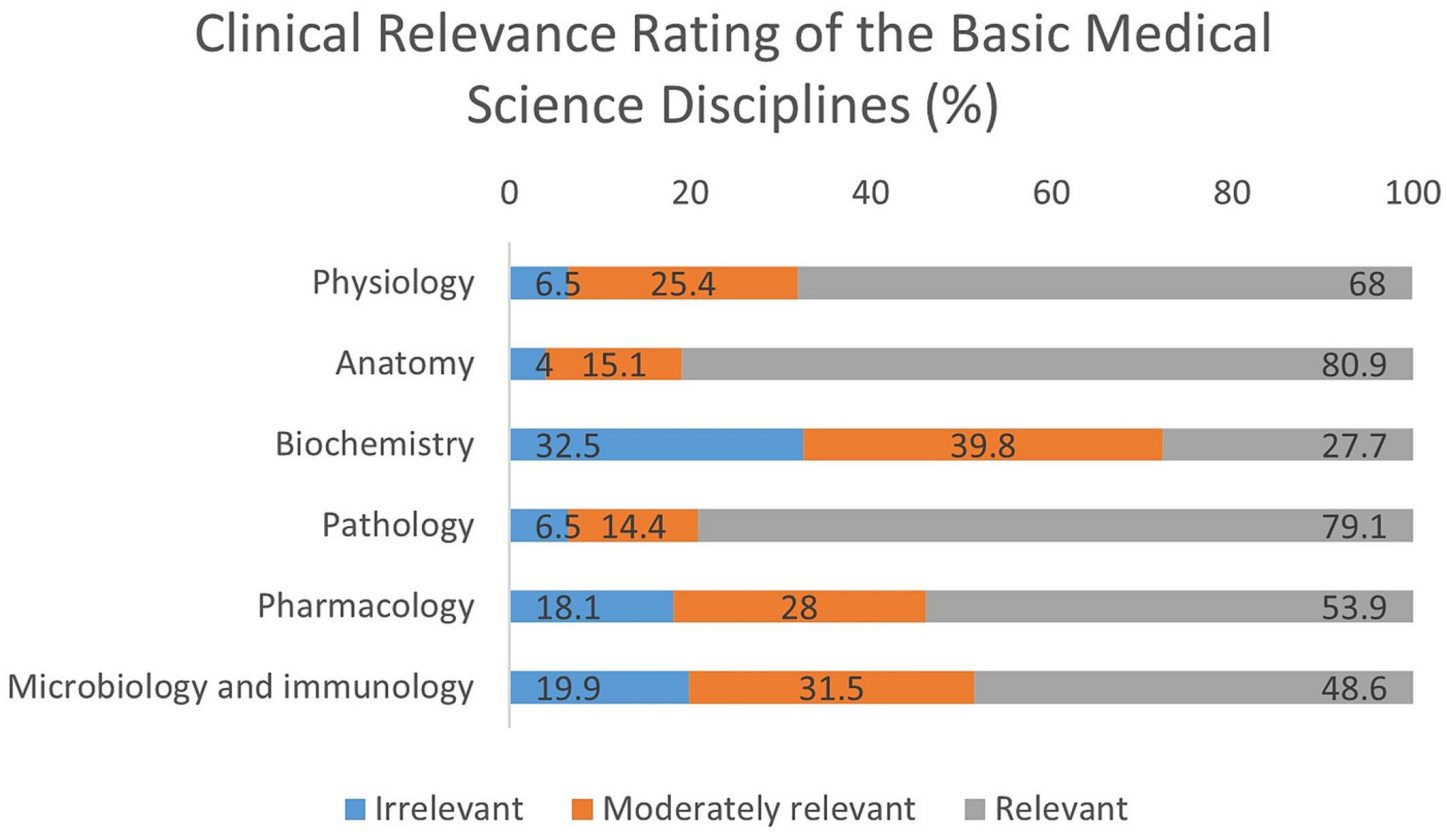
Distribution of student’s responses about the clinical relevance of the various basic medical sciences disciplines (%).

Figure 1 and Supplementary Table S4 show the clinical relevance rating of the basic medical science disciplines. Anatomy and pathology emerged as the most clinically valued disciplines, with 80.9 and 79.1% of students, respectively, rating them as relevant. Only a small fraction found these subjects irrelevant (4.0% for anatomy and 6.5% for pathology), indicating a strong recognition of their practical importance in clinical settings. Physiology was also highly rated, with 68.0% of participants considering it relevant and 25.4% finding it moderately relevant. Only 6.5% viewed it as irrelevant, reinforcing its foundational role in understanding clinical mechanisms. In contrast, biochemistry received the lowest relevance rating, with only 27.7% perceiving it as relevant. A significant portion of students (32.5%) rated it as irrelevant, and the majority (39.8%) considered it moderately relevant. This may reflect challenges in connecting biochemical concepts directly to clinical practice, highlighting a potential area for curriculum enhancement.

Pharmacology was rated as relevant by over half of the respondents (53.9%), though a considerable proportion (28.0%) rated it moderately relevant and 18.1% as irrelevant, suggesting variability in how well students link pharmacological knowledge with clinical application. Similarly, microbiology and immunology were viewed as relevant by 48.6% of participants, with 31.5% rating them as moderately relevant and 19.9% as irrelevant. These mixed perceptions may be influenced by how these subjects are taught or integrated into clinical learning.

### Students’ collective perception and attitude toward basic medical sciences

**Figure 2:**
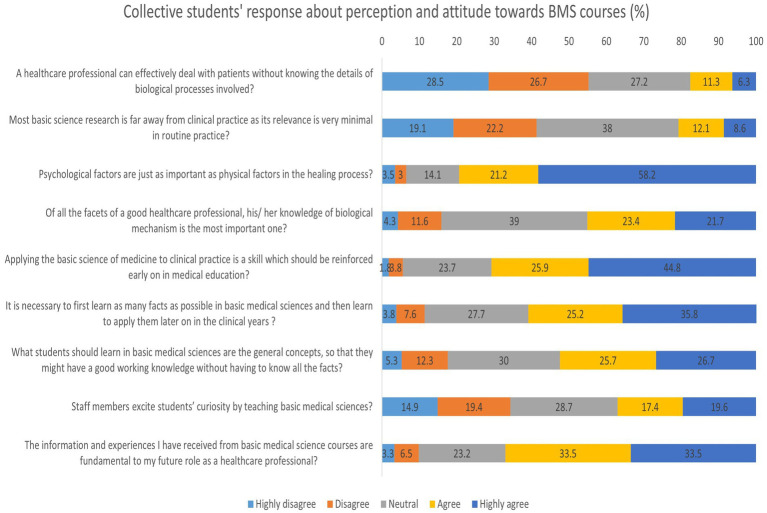
Distribution of student’s responses regarding the perception and attitude toward basic medical science courses (%).

The collective response of the students regarding their perception and attitude toward BMS is given in Figure 2 and Supplementary Table S5. A significant majority of students disagreed (28.5% highly disagree, 26.7% disagree) with the notion that healthcare professionals can manage patients effectively without understanding the underlying biological processes (median = 2.00). This reinforces the importance students place on foundational biomedical knowledge in clinical settings.

Regarding the perceived gap between basic science research and clinical practice, responses were more varied (median = 3.00), with 38.0% remaining neutral. While 41.3% expressed disagreement (either strongly or moderately), a combined 20.7% agreed to some extent, indicating that some students perceive a disconnect between research and real-world practice. An overwhelming 79.4% agreed or strongly agreed that psychological factors are just as important as physical ones in the healing process (median = 5.00), showing a holistic understanding of patient care among the respondents. While this item was retained from the original validated scale, it reflects a broader biopsychosocial perspective on healthcare rather than attitudes specifically toward BMS. This finding is therefore presented as an exploratory observation, suggesting students possess a holistic understanding of patient care.

Responses to whether knowledge of biological mechanisms is the most important aspect of a good healthcare professional were split (median = 3.00), with 39.0% neutral and a near-equal number expressing agreement (45.1%) and disagreement (15.9%). This suggests that while biological knowledge is valued, students may also consider other skills equally important in clinical competence. Strong support was observed for integrating BMS into early clinical education, with 70.7% agreeing or strongly agreeing that applying basic science in clinical practice should be reinforced early (median = 4.00). Similarly, 61.0% believed it is necessary to first learn factual knowledge in BMS before clinical application (median = 4.00), indicating a preference for a structured, stepwise approach to medical education.

When asked whether understanding general concepts is sufficient over memorizing exhaustive facts, responses were relatively balanced (median = 4.00), with 52.4% agreeing or strongly agreeing. This reflects a shift in learning preference toward conceptual understanding rather than rote memorization. However, perceptions of how engaging BMS teaching is were more mixed. While 37.0% agreed that staff members excite curiosity through teaching, 34.3% disagreed, and 28.7% remained neutral (median = 3.00). This highlights a potential area for faculty development in making BMS content more engaging and inspiring. Finally, the perceived utility of BMS education was high, with 67.0% agreeing that BMS knowledge is fundamental to their future roles as healthcare professionals (median = 4.00), reinforcing the enduring relevance of BMS in the professional identity formation of medical students.

### Student perceptions and attitudes toward the basic medical sciences curriculum

**Figure 3:**
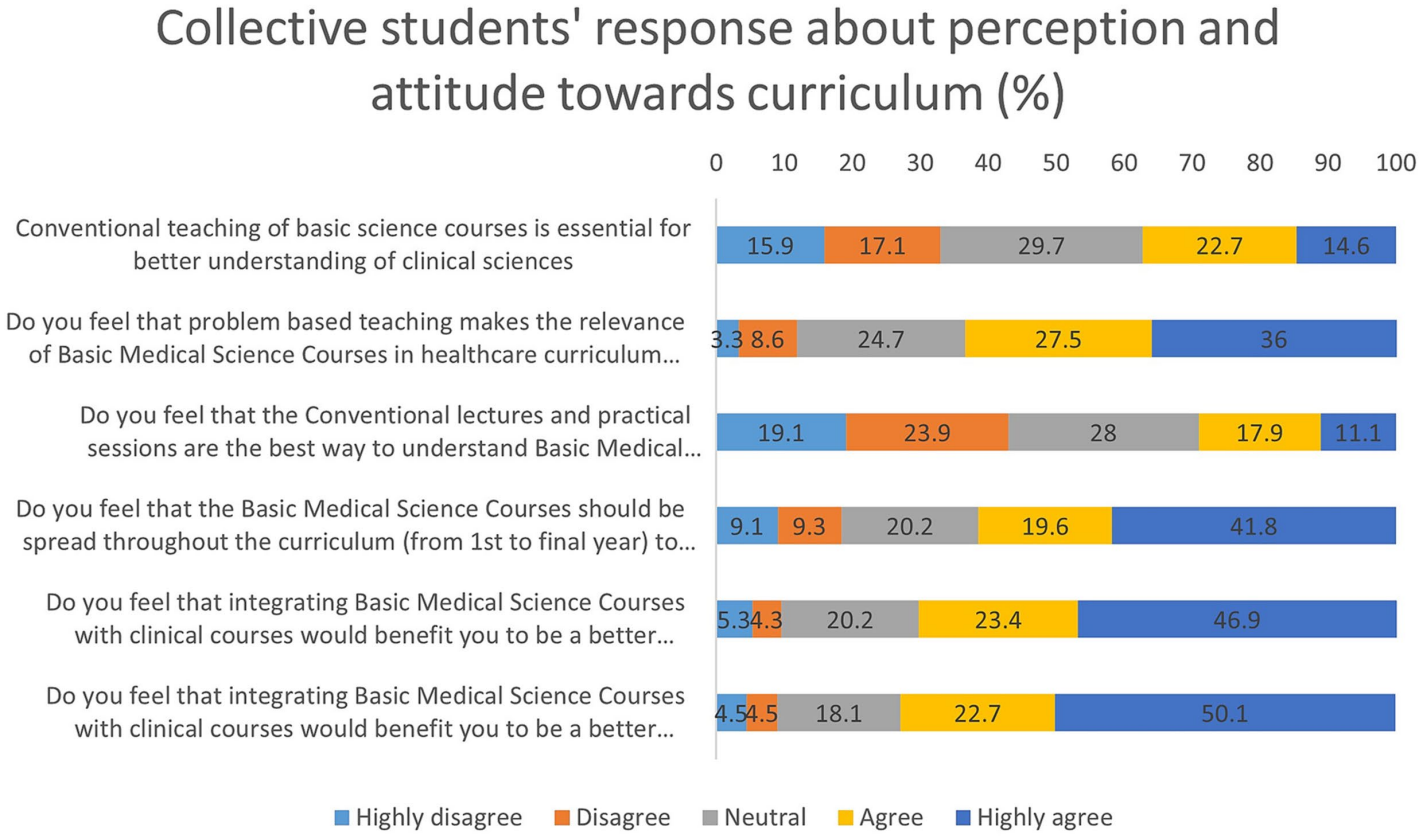
Distribution of student’s responses on the perception and attitude toward the curriculum (%).

The collective response of the students regarding their perception and attitude toward their curriculum is given in Figure 3 and Supplementary Table S6. When asked whether conventional teaching methods are essential for understanding clinical sciences, responses were mixed (median = 3.00). About 32.0% agreed or strongly agreed, while 33.0% disagreed or strongly disagreed, and 29.7% remained neutral. This suggests a lack of consensus among students on the effectiveness of traditional lectures and practicals in linking basic sciences to clinical application.

In contrast, there was strong support for problem-based teaching, with 63.5% of students agreeing or strongly agreeing that such an approach enhances the relevance of BMS in the healthcare curriculum (median = 4.00). This indicates a preference for active learning strategies that contextualize basic science content within clinical scenarios.

Regarding the statement that conventional lectures and practicals are the best way to understand BMS, only 29.0% of students agreed, while 43.0% disagreed, and 28.0% remained neutral (median = 3.00). This further underscores the shift in student preferences toward more interactive and integrated methods of teaching. A notable majority (61.4%) agreed or strongly agreed that spreading BMS courses throughout the curriculum, rather than concentrating them in the early years, would reduce knowledge overload and enhance learning retention (median = 4.00). This suggests that students are receptive to a longitudinal integration of BMS with clinical subjects across all years of study.

Integration of BMS with clinical courses was perceived positively. About 70.3% of students agreed or strongly agreed that such integration would make them better healthcare professionals (median = 4.00), and an even higher proportion (72.8%) believed it would benefit them as researchers (median = 5.00). These findings reflect a strong student endorsement of vertical integration in curriculum design, linking foundational sciences with both clinical and research competencies.

### Influence of socio-demographic variables on students’ perceptions and attitudes toward basic medical sciences

The subgroup analysis explored variations in students’ responses based on sex, curriculum type, program of enrollment, university type, age, and year of study to assess how these demographic and academic variables influence perceptions and attitudes toward BMS (Table 3 and Supplementary Table S7).

**Table 3:** Subgroup comparison of the students’ response about perception and attitude toward BMS.

Question	Gender			Type of curriculum				Program enrolled						Type of university		
	Male mean rank (*N* = 207)	Female mean rank (*N* = 190)	*p*-value	Conventional curriculum – (Individual course based) (*N* = 225)	Problem based curriculum (*N* = 141)	Others mean rank (*N* = 31)	*p*-value	Medicine mean rank (*N* = 101)	Dentistry mean rank (*N* = 179)	AMS mean rank (*N* = 41)	Nursing mean rank (*N* = 48)	Other healthcare mean rank (*N* = 28)	*p*-value	Public mean rank (*N* = 354)	Private mean rank (*N* = 43)	*p*-value
Q1	207.54	189.69	0.085	202.91	190.72	208.27	0.476	215.18	198.49	202.78	180.89	169.43	0.175	196.13	222.63	0.111
Q2	203.50	194.10	0.381	194.55	203.89	209.10	0.618	205.71	197.25	212.63	199.50	165.18	0.416	196.30	221.23	0.148
Q3	191.13	207.58	**0.043**	198.80	204.34	176.19	0.215	192.46	202.02	190.76	202.66	209.11	0.760	201.94	174.84	**0.038**
Q4	198.86	199.15	0.979	206.88	187.69	193.23	0.226	167.50	215.83	197.12	195.90	213.07	**0.007**	197.21	213.77	0.331
Q5	189.33	209.54	**0.027**	203.45	197.26	174.63	0.247	189.29	205.50	196.46	197.20	199.29	0.717	200.23	188.90	0.442
Q6	200.89	196.94	0.692	195.86	215.23	147.95	**0.002**	182.88	207.08	200.77	198.76	203.32	0.419	199.41	195.62	0.813
Q7	204.64	192.85	0.260	187.48	219.00	191.65	**0.017**	204.06	203.29	186.71	180.41	203.18	0.611	199.55	194.47	0.762
Q8	191.68	206.97	0.159	206.69	182.11	220.00	0.056	190.40	201.78	195.51	203.60	209.45	0.886	200.51	186.57	0.424
Q9	197.32	200.83	0.714	191.99	215.89	173.10	**0.018**	186.30	203.47	179.10	217.02	214.45	0.167	200.38	187.64	0.406

Comparison between subgroups done using Mann–Whitney *U* and Kruskal–Wallis test. Significant *P*-value (*P* < 0.05) given in bold.

Significant sex-based differences were noted in a few areas. Female students had significantly higher agreement levels than males for statements related to the importance of psychological factors in healing (Q3; *p* = 0.043) and the reinforcement of basic science applications early in medical education (Q5; *p* = 0.027). This can be considered an exploratory finding.

Students enrolled in problem-based curricula rated the relevance of integrating BMS into clinical teaching (Q7) and the application of BMS knowledge in early clinical training (Q9) significantly higher than those in conventional or other curricula (*p* = 0.017 and *p* = 0.018, respectively).

Students from different healthcare programs showed significant differences in their responses to specific questions. For instance, Q4 (“It is necessary to first learn facts in BMS before applying them clinically”) revealed a statistically significant variation among programs (*p* = 0.007), with medical students ranking this item lower than students from allied health and nursing backgrounds.

Students from public universities showed significantly higher agreement for Q3 than those from private institutions (*p* = 0.038), indicating potential differences in how psychological aspects of care and BMS are emphasized across institutional settings.

Supplementary Table S7 shows that no significant differences were observed across age groups or years of study for any of the nine perception-based questions (*p* > 0.05). This uniformity suggests that core perceptions toward BMS education are relatively stable throughout the academic journey and across age groups.

### Influence of socio-demographic variables on students’ perceptions and attitudes toward the curriculum

The subgroup comparison evaluated variations in students’ responses related to their perceptions and attitudes toward the healthcare curriculum, with respect to sex, curriculum type, healthcare program, university type, age, and year of study (Table 4 and Supplementary Table S8).

**Table 4:** Subgroup comparison of the students’ response about perception and attitude toward curriculum.

Question	Gender			Type of curriculum				Program enrolled						Type of university		
	Male mean rank (*N* = 207)	Female mean rank (*N* = 190)	*p*-value	Conventional curriculum – (Individual course based) (*N* = 225)	Integrated-Problem based curriculum (*N* = 141)	Others mean rank (*N* = 31)	*p*-value	Medicine mean rank (*N* = 101)	Dentistry mean rank (*N* = 179)	AMS mean rank (*N* = 41)	Nursing mean rank (*N* = 48)	Other healthcare mean rank (*N* = 28)	*p*-value	Public mean rank (*N* = 354)	Private mean rank (*N* = 43)	*p*-value
Q1	187.32	211.73	**0.025**	233.46	143.50	201.27	**<0.001**	204.88	199.89	190.57	188.15	203.04	0.897	197.54	211.03	0.439
Q2	194.56	203.84	0.345	175.77	243.08	167.15	**<0.001**	189.82	204.74	201.13	191.66	204.89	0.755	199.33	196.27	0.846
Q3	195.04	203.31	0.443	223.06	156.63	217.06	**<0.001**	204.72	204.37	180.98	188.27	188.79	0.622	196.39	220.51	0.164
Q4	206.96	190.33	0.097	179.76	230.53	195.23	**<0.001**	188.26	199.38	185.50	230.41	201.25	0.152	199.92	191.47	0.599
Q5	202.29	195.42	0.457	192.94	211.80	184.71	0.108	189.96	195.25	192.39	229.36	213.21	0.118	198.75	201.05	0.877
Q6	199.82	198.10	0.848	190.84	215.37	183.73	**0.024**	187.07	200.07	210.74	209.39	200.16	0.528	198.30	204.74	0.656

Comparison between subgroups done using Mann–Whitney *U* and Kruskal–Wallis test. Significant *P*-value (*P* < 0.05) given in bold.

A statistically significant difference was found for Q1 (*p* = 0.025), where female students showed a more favorable response regarding the essential role of conventional teaching in understanding clinical sciences. This suggests that female students may place more value on structured and traditional educational approaches compared to their male counterparts.

Highly significant differences were observed across most questions when comparing curriculum types. Students from problem-based curricula consistently reported more favorable perceptions in key areas:

*Q1*: Importance of conventional teaching (*p* < 0.001).

*Q2*: Impact of problem-based learning on the relevance of BMS (*p* < 0.001).

*Q3*: Effectiveness of conventional lectures and practical sessions (*p* < 0.001).

*Q4*: Integration of BMS throughout the curriculum (*p* < 0.001).

*Q6*: Integration of BMS with clinical content to benefit research (*p* = 0.024).

These findings reinforce that students in problem-based curricula may better appreciate integrated and longitudinally structured medical education models that bridge basic and clinical sciences.

No significant differences were observed across programs, types of university, age groups, and years of study (*p* > 0.05).

### Determinants of positive and negative attitudes toward basic medical sciences

Students’ positive attitude toward basic medical sciences was significantly influenced by the type of curriculum and year of study. Those enrolled in a PBL-integrated curriculum showed a higher positive attitude (*B* = 0.220, *p* = 0.007) compared to students from other curriculum types. Third-year students demonstrated a significantly lower positive attitude compared to interns (*B* = −0.151, *p* = 0.048). No other sociodemographic factors showed a significant association with a positive attitude toward BMS.

**Figure 4:**
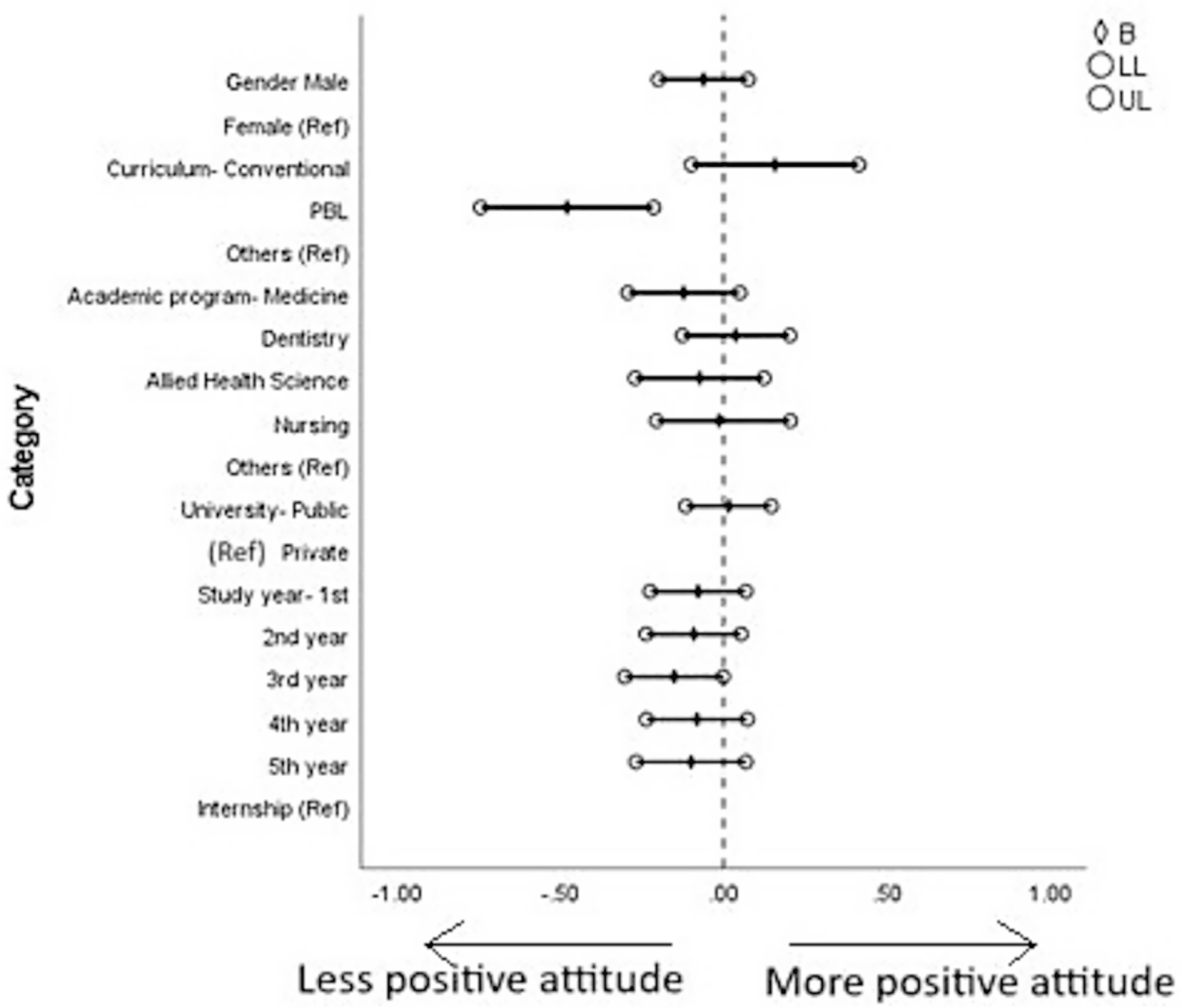
Forest plot showing multiple linear regression analysis of positive attitudes toward basic medical science courses. B, beta; LL, lower limit of confidence interval; UL, upper limit of confidence interval. The results of this regression model are tabulated in Supplementary Table S9.

**Figure 5:**
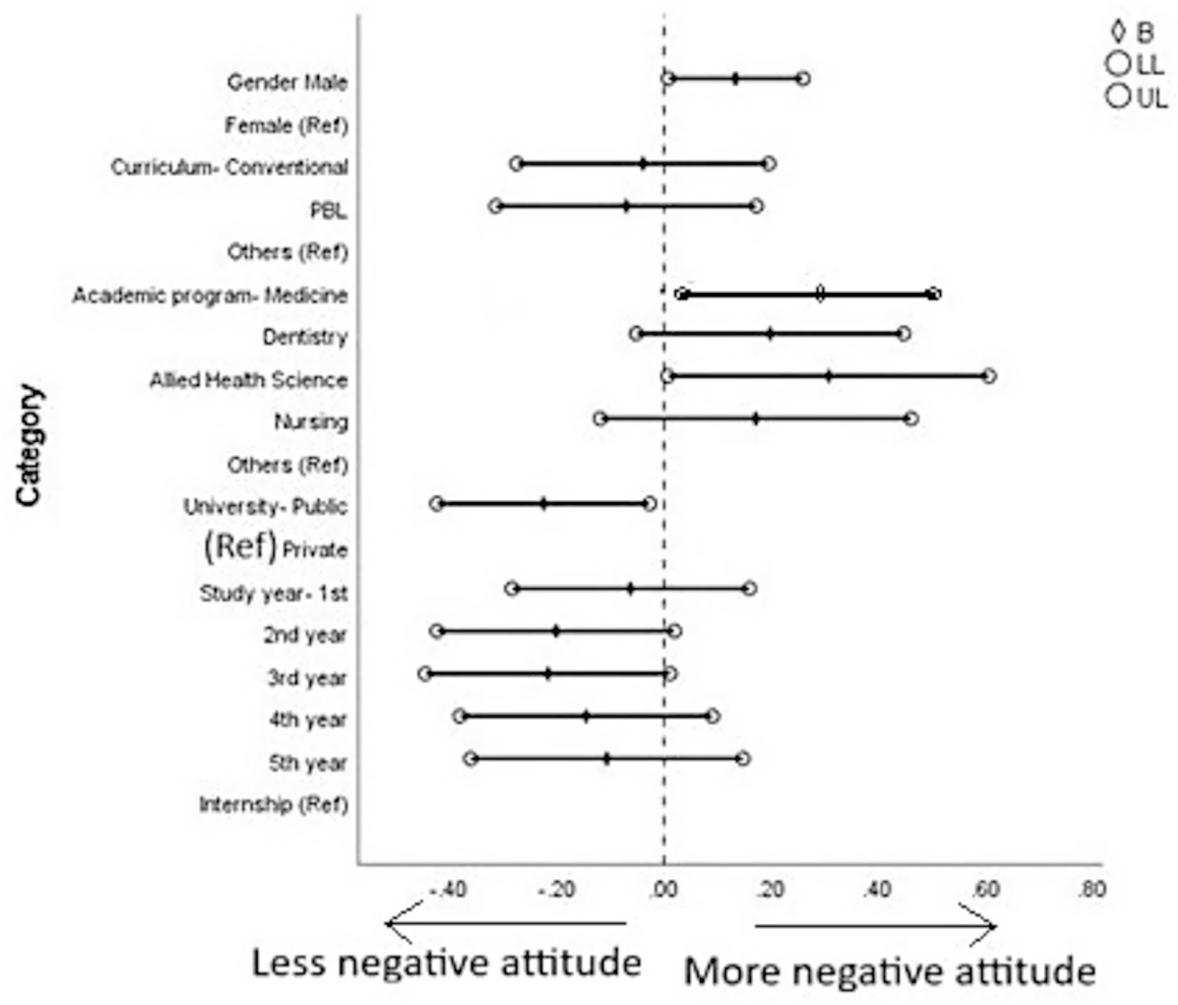
Forest plot showing multiple linear regression analysis of negative attitudes toward basic medical science courses. B, beta; LL, lower limit of confidence interval; UL, upper limit of confidence interval. The results of this regression model are tabulated in Supplementary Table S9.

Students’ negative attitude toward basic medical sciences was significantly associated with sex, academic program, and type of university. Male students had a higher negative attitude compared to females (*B* = 0.134, *p* = 0.036). Students from medicine (*B* = 0.296, *p* = 0.026) and allied health sciences (*B* = 0.308, *p* = 0.044) programs exhibited higher negative attitudes compared to those from other programs. Additionally, students from public universities demonstrated a lower negative attitude compared to those from private universities (*B* = −0.224, *p* = 0.027). No other significant associations were observed (Figures 4, 5 and Supplementary Table S9).

### Determinants of attitudes toward curricula

Students’ attitude toward the conventional curriculum was significantly associated with the type of curriculum. Students following a PBL-integrated curriculum reported a significantly lower positive attitude toward the conventional curriculum (*B* = −0.479, *p* < 0.001) compared to students from other curriculum types. No other sociodemographic factors showed a significant association.

**Figure 6:**
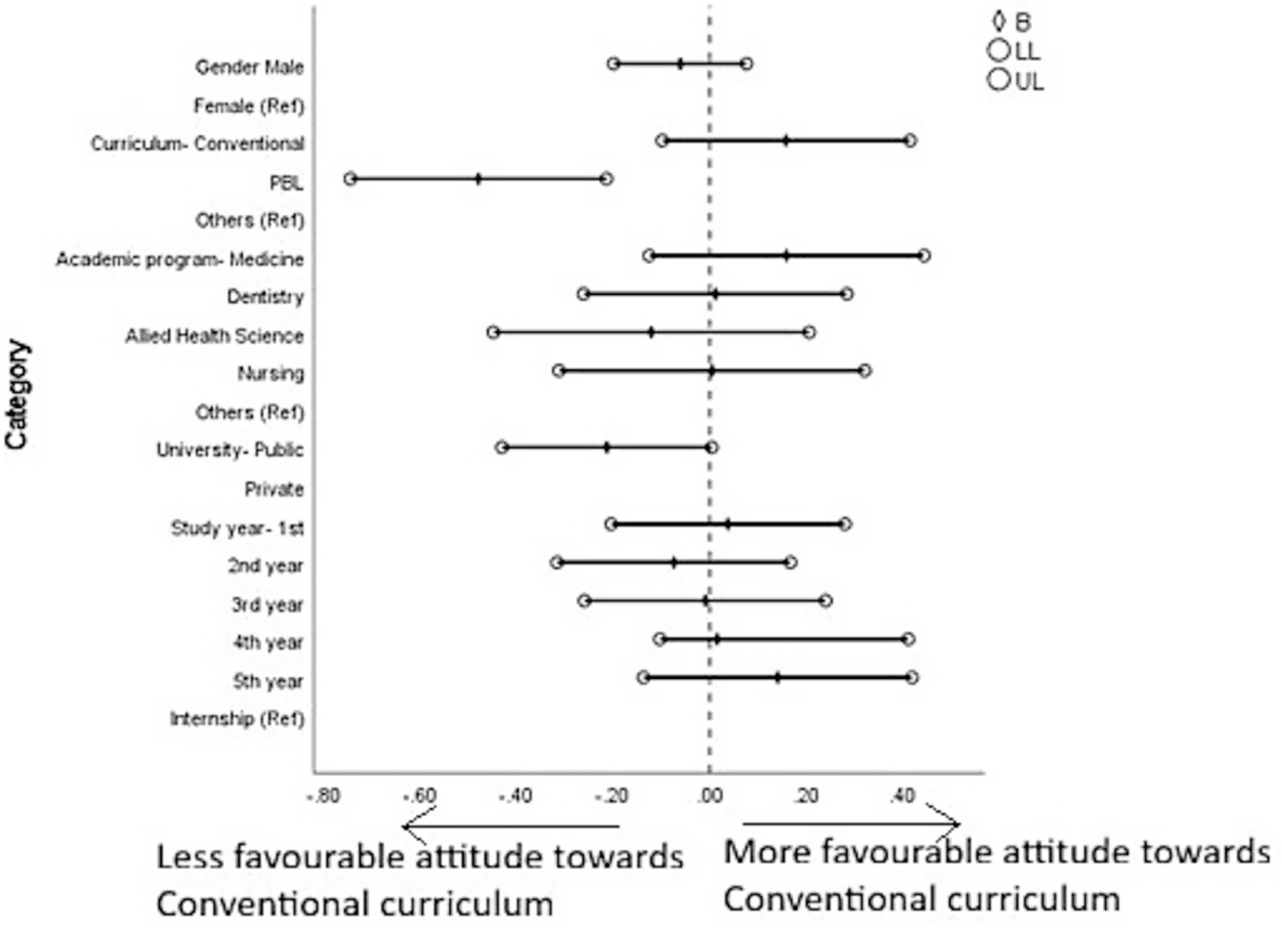
Forest plot showing multiple linear regression analysis of attitudes toward conventional curriculum. B, Beta; LL, lower limit of confidence interval; UL, upper limit of confidence interval. The results of this regression model are tabulated in Supplementary Table S10.

**Figure 7:**
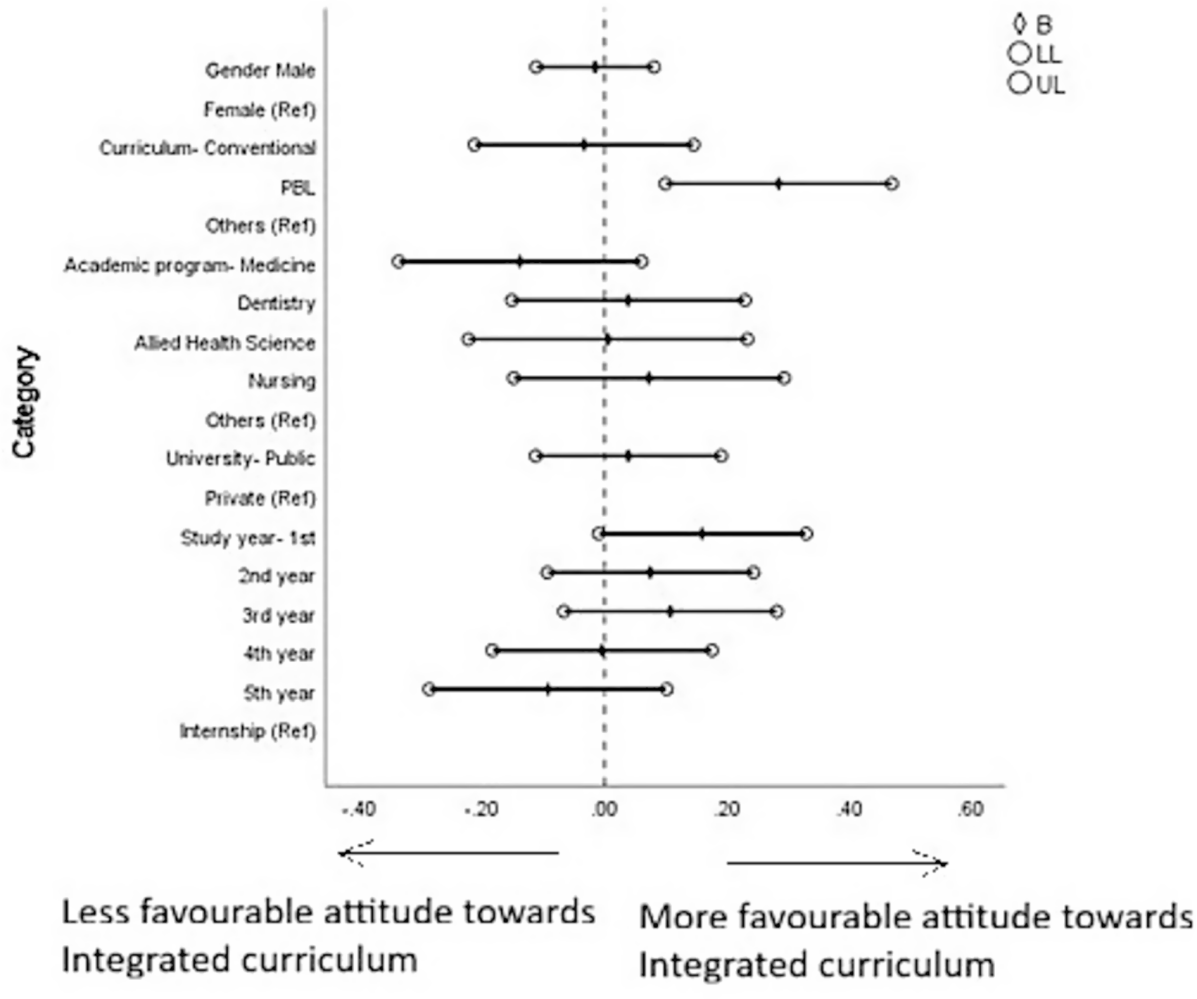
Forest plot showing multiple linear regression analysis of attitudes toward the integrated curriculum. B, beta; LL, lower limit of confidence interval; UL, upper limit of confidence interval. The results of this regression model are tabulated in Supplementary Table S10.

Students’ attitude toward the PBL-integrated curriculum was also significantly influenced by the type of curriculum. Students enrolled in a PBL-integrated curriculum exhibited a significantly higher positive attitude toward the integrated curriculum (*B* = 0.285, *p* = 0.003). Other sociodemographic variables did not show any significant association (Figures 6, 7 and Supplementary Table S10).

### Interrelationship between students’ perceptions of BMS and attitude toward curriculum

Students’ positive perception toward BMS was positively correlated with both attitude toward the conventional curriculum and that of the PBL-integrated curriculum, while the former was weak (*r* = 0.154, *p* = 0.002) and the latter was moderate (*r* = 0.366, *p* < 0.001). A weak positive correlation was observed between negative perception toward BMS and attitude toward conventional curricula (*r* = 0.110, *p* = 0.029). Additionally, attitude toward PBL-integrated curricula was weakly and negatively correlated with attitude toward conventional curricula (*r* = −0.149, *p* = 0.003) (Table 5).

**Table 5:** Correlation between students’ attitude toward BMS and curriculum.

Factors	Positive perception toward BMS	Negative perception toward BMS	Attitude toward conventional curricula	Attitude toward integrated-problem based curriculum
Positive perception toward BMS	1.000	–	–	–
Negative perception toward BMS	**−0.133 (*p* = 0.008)**	1.000	-	-
Attitude toward conventional curricula	**0.154 (*P* = 0.002)**	**0.110 (*P* = 0.029)**	1.000	-
Attitude toward integrated curricula	**0.366 (*P* < 0.001)**	−0.086 (*P* **=** 0.086)	**−0.149 (*P* = 0.003)**	1.000

Correlation between attitudes and perception done using Spearman’s correlation test. The results are expressed as ρ (rho) value. *P* < 0.05 considered statistically significant (in bold).

## Discussion

Basic medical sciences (BMS) form the foundational framework upon which clinical knowledge and practice are built. A firm grasp of BMS concepts is essential not only for understanding the mechanisms of health and disease but also for developing competent healthcare professionals (1). The current study explored the perceptions and attitudes of healthcare students toward BMS and curriculum structure, analyzing overall trends and differences across various subgroups.

### General perceptions, attitudes, and interests toward basic medical sciences

The results regarding students’ perceptions and interest in basic medical sciences show a clear enthusiasm for the subject, yet with notable uncertainty about pursuing a BMS-related career. While 56.7% of students expressed interest in BMS courses, only 26.2% admitted interest in a career in the field. This enthusiasm–career gap can be interpreted through the lens of expectancy–value theory, which posits that career choices are shaped not only by intrinsic interest but also by perceived utility value, attainment value, and anticipated costs (64). Although students may find BMS intellectually stimulating (high intrinsic value), the perceived limited financial rewards (10.6%) and lack of excitement (14.4%) (Table 2) suggest that the utility and attainment values associated with BMS careers remain low. Similarly, social cognitive career theory emphasizes that self-efficacy beliefs and outcome expectations are critical determinants of career choice; students who lack exposure to successful BMS career role models may develop low outcome expectations for this pathway (65). This aligns with previous studies that found a general attraction to medical sciences in academic settings but a significant drop in students’ career intentions (34, 41, 66). The hesitation to pursue BMS careers was also evident in students’ reluctance to guide juniors toward the field. Only 19.4% of students expressed a willingness to encourage others to pursue BMS, a sentiment shared by other studies (41). This could reflect the lack of mentorship structures or role models in BMS, which is often a significant motivator for career decisions in the medical field (67, 68).

Moreover, the perception that BMS lacks excitement (14.4%) and offers limited financial benefits (10.6%) mirrors trends observed in other studies, where students expressed concerns about the lack of career rewards in academic disciplines versus clinical fields with more visible career progression and financial incentives (41, 69, 70), suggesting that students may be attracted to BMS as an academic subject but are hesitant about its professional appeal. Institutional investment in career development infrastructure may attenuate the enthusiasm–career gap (71). In the Saudi context, the absence of such structured pathways may partially explain why high academic interest fails to translate into career commitment.

Regarding curriculum design, the mixed responses about integrated learning reflect a larger ongoing debate in medical education. While 43.6% of students believed that a PBL-integrated curriculum would increase interest in BMS, 44.1% reported being unsure. This uncertainty may partly be explained by contextual factors: a majority of respondents were either not enrolled in PBL-integrated curricula (64.5%) or were still in the early years of study (43.4% in years 1–2), which may limit their ability to fully appreciate the potential benefits of curricular design. It was also noted that junior students, with limited clinical exposure, often find it difficult to evaluate the value of integration compared to their senior peers (72). Similar studies indicate that students often favor traditional, subject-specific approaches, but they acknowledge the need for more integrated learning to better connect theory with clinical practice (41, 73). The preference for a more integrated curriculum may therefore depend not only on exposure but also on how well students understand the long-term benefits of such a model, both academically and professionally.

The interest in research opportunities further emphasizes the need for more hands-on experiences in BMS, as 56.7% of students expressed interest in preclinical or animal research. This aligns with studies that suggest practical experience, particularly in research, can significantly enhance student engagement and career orientation in scientific fields (74). By offering more opportunities for students to engage with research, educational institutions could increase student interest in pursuing BMS careers by demonstrating the relevance of the subject to real-world clinical applications.

Interestingly, however, some students still harbored neutral or skeptical views, particularly on questions concerning the depth and necessity of basic science knowledge (Figure 2). For example, 17.6% of respondents agreed that a healthcare professional could manage patients without knowing the biological processes involved. This varied among similar studies carried out in other countries; around 41.4% of Jordanian medical students, 5% of clinical grade students, and 7.5% of pre-clinical grade students in a study conducted in Egypt shared the same perception (39, 75). Comparable findings have also been reported elsewhere: for instance, studies from Ethiopia and Pakistan noted that students often valued clinical skills more highly than foundational sciences (41, 66), while European cohorts expressed concern about the limited day-to-day applicability of BMS knowledge in clinical settings (70). This suggests that doubts about the practical value of BMS are common across countries, but the intensity varies—in our Saudi cohort, the skepticism was less pronounced comparatively, yet it still points to a gap in how the relevance of BMS is being communicated to students. Such differences likely reflect contextual influences, including curricular design, teaching approaches, and the degree of early clinical exposure. It may also reflect the perception among some students that practical, patient-facing skills outweigh theoretical knowledge, especially in systems where BMS is taught in a highly compartmentalized or didactic fashion. On the other hand, responses to statements such as “Psychological factors are just as important as physical factors in the healing process” (79.4%) (exploratory item) and “Applying the basic science of medicine to clinical practice should be reinforced early on” (70.7%) received high levels of agreement, with over 70% of students agreeing or strongly agreeing. This indicates a growing awareness among students of the biopsychosocial model of health, as well as the value of early integration of BMS, in line with findings from Woods et al. (76) and Norman (77), which support the educational benefits of early contextualization.

Thus, our findings show a clear pattern: while 56.7% of Saudi students expressed enthusiasm for learning BMS, only 26.2% indicated interest in a BMS-related career, and just 19.4% would recommend the field to juniors. This mirrors global trends of high academic interest but low career uptake (34, 41, 70). However, unlike Malaysian and Jordanian cohorts, where integrated curricula and applied exposure were associated with more favorable perceptions (39, 69), Saudi students appeared more ambivalent. For instance, 44.1% of our respondents were unsure whether a PBL-integrated curriculum would enhance BMS interest, despite 43.6% agreeing it could. This contrasts with clearer positive responses in settings where integration was firmly embedded. Similarly, while 56.7% of Saudi students expressed interest in research, the lack of structured research pathways may explain why this did not translate into stronger career intentions. However, unlike Ethiopian and Pakistani contexts where infrastructure limitations dominate career barriers (41, 66), Saudi students’ emphasis on financial concerns and lack of excitement suggests that attitudinal and motivational factors, rather than structural constraints, require prioritization in intervention design. Taken together, these findings suggest that Saudi students share the global pattern of valuing BMS as a subject but are uniquely constrained by mixed curricular models and underdeveloped research opportunities, highlighting the need for targeted interventions that address not only academic interest but also career self-efficacy and outcome expectations.

### Perception toward the clinical relevance of BMS

The perception of clinical relevance among Basic Medical Science (BMS) disciplines offers critical insights into how students value foundational knowledge in relation to their future clinical practice. The observed trend of high relevance assigned to anatomy, pathology, and physiology mirrors findings in prior research conducted across various educational contexts (39, 69, 75). Patel and Moxham (78, 79) emphasize anatomy’s direct application in physical examinations, while Pabst et al. (80) highlight how anatomical and pathological knowledge supports diagnostic reasoning. Studies indicate that students often view these subjects as directly applicable to clinical tasks such as diagnostics, physical examinations, and understanding disease processes, which likely accounts for their consistently favorable ratings across student groups (81, 82).

In contrast, biochemistry and microbiology/immunology were perceived as less relevant to clinical practice. This pattern is consistent with literature reporting that students often struggle to see the practical value of these subjects, especially when content is delivered in a highly theoretical or fragmented manner (42, 83, 84). For instance, a survey of medical students’ preferences for effective teaching found that students highly value clarity and relevance in instruction, suggesting a preference for subjects with direct clinical applicability (85). Biochemistry’s heavy molecular focus and limited integration with clinical examples may contribute to its lower perceived utility (83). Similarly, microbiology and immunology, despite their foundational role in understanding infections and immune responses, may not feel clinically connected without practical application during preclinical training. Interestingly, our analysis showed a weak but statistically significant positive correlation between year of study and perceived clinical relevance of biochemistry (*ρ* = 0.099, *p* = 0.048), suggesting that senior students may gradually recognize its clinical utility when biochemical principles are reinforced during clinical exposure. No such trends were observed for the other basic medical sciences, highlighting the continued need for integrative teaching approaches to demonstrate their role in infection management and immune-related conditions (Supplementary Table S11).

Pharmacology, while moderately rated in this study, has shown variable perceptions across different educational settings (39, 69, 75). Some literature attributes this to differences in how early and effectively pharmacological concepts are introduced in a clinical context (44, 86). For instance, in settings where pharmacology is reinforced through clinical case discussions or prescription-based simulations, students tend to perceive it as more relevant (87, 88).

Taken together, the differences across subjects can be explained by the degree of clinical integration and reinforcement during training. Highly applied subjects such as anatomy and pathology are valued from the outset, whereas the appreciation of biochemistry appears to emerge later with clinical exposure. While Jordanian and Egyptian students showed similar subject preferences (39, 75), the degree of dissatisfaction with biochemistry in our cohort (32.5% rating it irrelevant) exceeded that reported in Malaysian integrated programs (69), where contextual teaching improved perceptions. This suggests that pedagogical approach, not merely subject content, determines perceived relevance and underlines the importance of contextualizing all BMS subjects through integrative, case-based teaching to help students recognize their relevance throughout the curriculum. From an EVT perspective, subjects perceived as directly applicable to clinical practice (anatomy, pathology, physiology) carry higher utility value, reinforcing engagement, whereas biochemistry’s lower perceived relevance reflects diminished utility value despite its foundational importance, thus highlighting the need to explicitly demonstrate its clinical applications. These differential perceptions of clinical relevance are not merely academic preferences; they may also have direct implications for how students evaluate curricular structures, which is evaluated in the next section.

### General perceptions, attitudes, and interests toward curricular structure

Students in this study showed a strong preference for a PBL-integrated BMS curriculum, both vertically and horizontally. The high level of agreement with the statement “Basic medical sciences should be distributed across all academic years” reflects a positive perception of vertical integration. This aligns with findings from Teshome et al. (41) and Althubaiti and Althubaiti (34), where students emphasized the importance of reinforcing foundational sciences throughout their training to better support clinical reasoning.

Similarly, students strongly endorsed horizontal integration, as seen in their support for teaching BMS subjects thematically—for example, integrating anatomy, physiology, pathology, and pharmacology around body systems. This preference corresponds with Harden’s (89) principles of curriculum integration and has been echoed in studies like those by Karim et al. (73) and Din et al. (66), where students reported higher engagement and understanding when content was organized around clinical themes.

The statement “Problem-based learning helps in understanding basic sciences better” also received substantial support, underscoring the value students place on active learning strategies. This is consistent with global research by Dolmans and Schmidt (90) and Prince et al. (91), which shows that PBL enhances critical thinking and improves knowledge retention. However, the level of uncertainty observed among Saudi students (44.1% unsure about integration benefits) contrasts with clearer endorsements in Malaysian cohorts (73%) and more pronounced dissatisfaction with conventional models in Ethiopian settings (43%). This preference aligns with EVT’s utility value construct, as integrated PBL contextualizes abstract BMS content within clinical scenarios, thereby enhancing perceived usefulness and reducing the psychological ‘cost’ of learning foundational sciences in isolation from patient care. However, it is important to note that the evidence for PBL’s superiority is not unequivocal. Colliver (92) found that effect sizes for PBL on knowledge acquisition were small and clinically insignificant, while Albanese (93) argued that such modest effects are to be expected given the complexity of educational interventions. In our study, the favorable perception of PBL may reflect student satisfaction and perceived learning benefit rather than objectively measured knowledge gains, a distinction that warrants further investigation through comparative outcome studies in Saudi educational settings. Similar preferences have been noted in studies where students favored PBL and case-based learning (CBL) for their ability to contextualize abstract scientific content within clinical scenarios (41, 94).

In contrast, student responses to “Lectures are the best way to understand basic medical sciences” were mixed. While some students still appreciated the structure and clarity lectures provided, especially for complex subjects, others found them less engaging. These findings are comparable to related studies where students advocated for a blended approach combining traditional lectures with interactive sessions to accommodate diverse learning preferences (95, 96).

Lastly, students expressed a strong interest in early exposure to clinical cases and research within the BMS curriculum. This supports similar findings from studies by Cuschieri and Cuschieri (74) and Querido et al. (70), which highlight the motivational role of early clinical exposure (ECE) and research involvement in enhancing the perceived relevance of BMS. These strategies not only reinforce learning but also encourage inquiry and deeper engagement with the material. This intermediate position may reflect Saudi Arabia’s curricular heterogeneity, where students lack consistent exposure to fully integrated models especially in non-MBBS programs. While these curricular preferences were broadly consistent across the sample, important variations emerged when examining how sociodemographic and academic factors shaped students’ attitudes, as discussed below.

### Sociodemographic determinants of students’ attitude toward BMS and curriculum

Subgroup analysis (Table 3 and Supplementary Table S6) and regression analysis (Figures 4, 5) revealed several patterns in students’ perceptions of basic medical sciences (BMS) based on demographic and academic variables. Female students showed significantly higher agreement with statements emphasizing holistic care and early integration of BMS, such as recognizing the importance of psychological factors in healing and the need to reinforce BMS concepts early in clinical practice. However, this item reflects a broader biopsychosocial perspective and is interpreted as an exploratory finding rather than a core indicator of BMS attitudes. These findings are consistent with prior research, which reported that female medical students often display stronger empathy and favor interdisciplinary and patient-centered learning models (97, 98). In addition, male students demonstrated a higher negative attitude toward BMS compared to female students, a trend that has been similarly observed in earlier research suggesting that female students often report higher motivation and academic satisfaction (99). The differences may also reflect sex-related variations in learning styles, with female students more attuned to contextual and integrative content (100). These sex-based and program-related differences are consistent with EVT’s recognition that socializers and contextual factors shape domain-specific value appraisals, suggesting that educational environments differentially influence how students perceive the value of BMS. Unlike Western contexts where sex differences are primarily attributed to empathy and learning style variations, the Saudi findings may additionally reflect the Kingdom’s recent educational reforms expanding female participation in healthcare, creating distinct professional expectations that shape how female students engage with foundational sciences.

When studying curriculum types, students enrolled in PBL-integrated curricula consistently demonstrated more favorable attitudes toward BMS, particularly in their support for early clinical application and the relevance of foundational sciences (Q6, 7, 9). Additionally, students following a problem-based learning curriculum exhibited a more positive attitude toward BMS compared to those from other curricula. This is in line with studies that found PBL enhances intrinsic motivation and encourages deeper learning by situating basic science concepts within real-world clinical contexts (101, 102). In contrast, students in conventional curricula were more likely to perceive BMS as isolated or disconnected from patient care—likely due to limited early clinical exposure and compartmentalized teaching methods (103). Differences also emerged based on academic programs: students from dental and medical fields exhibited a stronger appreciation for the application of BMS (Q4), possibly due to the more rigorous and extensive basic science training in their curricula. In contrast, students in nursing and allied medical sciences programs showed more neutral or varied responses, which may reflect the comparatively reduced emphasis on BMS in skill-focused or practice-oriented training environments (42). In contrast, students enrolled in medicine and allied health sciences exhibited more negative attitudes toward BMS compared to students in other programs. This could be attributed to greater academic pressures and perceived curriculum overload in these programs, making basic sciences seem burdensome rather than foundational (30). While differences based on university type were not uniformly significant, students from private institutions tended to show slightly higher agreement with items linking BMS to psychosocial and clinical aspects (Q3). As this item addresses psychosocial aspects of healing rather than core BMS content, this observation is noted as exploratory and may reflect institutional differences in emphasis on holistic care perspectives. This suggests that private universities may offer more supportive learning environments, characterized by smaller class sizes, better student–teacher interactions, and more innovative teaching strategies (104). Interestingly, students from public universities showed lower negative attitudes than those from private universities, possibly reflecting differences in curricular design, teaching quality, or institutional culture that could influence student perceptions. Interestingly, no statistically significant variation was found across different age groups or academic years, indicating that attitudes toward BMS remain relatively stable throughout the educational trajectory. This differs from earlier research by Saleh and Adley (105), which observed a decline in interest in BMS during later years due to the increasing focus on clinical practice. However, third-year students showed a lower positive attitude toward BMS compared to interns. A possible explanation could be that mid-program students are often heavily involved in clinical exposure, which might shift their focus away from basic sciences, perceiving them as less immediately relevant (105). In contrast, interns, having completed both basic and clinical training, may better appreciate the foundational role of BMS in clinical practice.

With regard to the determinants of students’ attitudes toward curricular structure, although students from PBL-integrated curricula showed an overall lower positive attitude toward the conventional curriculum and a higher positive perception of the PBL-integrated curriculum, they still reported favorable perceptions regarding specific aspects such as the importance of conventional teaching (cQ1) and the effectiveness of lectures and practical sessions (cQ3). This apparent contradiction suggests that while students from PBL-integrated curricula prefer integrated, clinically oriented learning, they still value the structured delivery and clarity that conventional methods offer for foundational knowledge (106). This apparent inconsistency may warrant a nuanced understanding that students from PBL-integrated curricula may still prefer foundational knowledge delivery through structured lectures and finds it valuable even within an integrated framework—a view aligned with constructivist learning theory, which posits that learners build new knowledge upon existing scaffolds (107). Similar findings have been reported in previous studies, where students in hybrid or PBL-integrated curricula acknowledged lectures as effective tools for organizing core concepts (108). Thus, preserving key elements of conventional teaching within PBL-integrated curricula may better align with student learning preferences. The significant association found in cQ6, where students from PBL-integrated curricula perceived better integration of BMS with clinical content to benefit research, further supports the argument that integrated curricula better prepare students for academic inquiry and translational research pathways. The lack of significant associations with other sociodemographic variables such as sex, year of study, and type of university suggests that the curriculum model itself is a primary determinant shaping students’ perceptions and attitudes, independent of individual background factors. This observation underscores the powerful influence of educational design on student mindset and satisfaction, a theme echoed in prior studies advocating for integrated and student-centered learning environments in medical education (109). These demographic and curricular influences on individual attitudes naturally raise the question of how BMS perceptions and curricular preferences interact at a broader level, which is examined in the following section.

### Interrelationship between students’ attitudes toward BMS and curricular structure

Finally, studying the interrelationship between students’ attitudes toward BMS and curricular structure showed interesting results. The positive perception of basic medical sciences (BMS) showed a stronger association with a favorable attitude toward PBL-integrated curricula than with conventional curricula. This suggests that students who appreciate the relevance of BMS tend to prefer integrated learning models that closely link basic and clinical sciences. Prior studies have also highlighted that integrated curricula enhance the perceived relevance and application of BMS in clinical practice (8, 110). Conversely, the weak positive correlation between negative BMS perception and preference for conventional curricula may reflect that dissatisfaction with BMS aligns more with traditional, discipline-based teaching approaches, which have been criticized for promoting fragmented knowledge and limited clinical applicability (103, 111).

### Implications of the study

The findings from this study offer key insights for healthcare curriculum design. Students across subgroups strongly favored vertical and horizontal integration of basic medical sciences (BMS), underscoring the need to embed BMS content throughout the academic journey rather than teaching it in isolation. Active learning strategies like problem-based learning and early clinical exposure were also well-received, highlighting their role in making BMS topics more engaging and clinically relevant. Program-specific variations suggest that BMS content should be tailored to each discipline’s needs, linking basic science to practical applications like patient care in nursing. Implementing these reforms will require ongoing faculty development to support integrated, student-centered teaching. Furthermore, the strong association between positive perceptions of BMS and preference for PBL-integrated curricula emphasizes the importance of connecting basic science to clinical practice to sustain student interest. Despite academic enthusiasm, hesitancy to pursue BMS-related careers points to the need for stronger mentorship, clearer career pathways, and early research opportunities. Finally, sex- and program-based differences call for more personalized, inclusive educational strategies to better meet diverse learning needs.

In contextual perspective, Saudi students occupy a distinctive position internationally. While sharing positive BMS attitudes with peers in Jordan, Egypt, and Malaysia (39, 69, 75), they demonstrate greater ambivalence about curricular preferences and career pathways. This ambivalence likely reflects the Kingdom’s transitional educational landscape characterized by variable integrated PBL adoption across institutions and programs, accreditation-driven reforms (37), and evolving professional identities (34, 35). These factors could distinguish the Saudi experience from both established integrated systems in Western contexts and predominantly conventional systems in South Asian settings.

Collectively, the findings of this study can be coherently interpreted through the expectancy–value theory framework. The discrepancy between high interest in BMS courses (56.7%) and low career intentions (26.2%) reflects the gap between intrinsic value and the utility/attainment values necessary for career commitment, with perceived costs such as viewing BMS as a less thrilling field (14.4%) and offering limited financial growth (10.6%) acting as deterrents. The differential clinical relevance ratings, with anatomy (80.9%) and pathology (79.1%) rated highest and biochemistry lowest (27.7%), demonstrate how utility value perceptions vary across BMS disciplines. The moderate positive correlation between positive BMS perceptions and support for integrated curricula (r = 0.366, *p* < 0.001) further supports EVT’s proposition that educational contexts enhancing perceived usefulness strengthen engagement. These patterns affirm the applicability of EVT for understanding healthcare students’ engagement with foundational sciences in the Saudi Arabian context.

### Strengths and limitations

The present study has several strengths. Firstly, it involved a diverse participant pool, including students from multiple healthcare disciplines (medicine, dentistry, nursing, applied medical sciences, etc.) and different academic years. This helped provide a broad and inclusive perspective on perceptions toward basic medical sciences. Additionally, the study incorporated subgroup comparisons based on sex, curriculum type, program enrolled, university type, age, and year of study, offering a more nuanced understanding of how various demographics perceive BMS and curriculum structure. Another strength was the use of a previously validated tool (57) for part of the questionnaire, adding credibility to the instrument design (57). The study also focused on the clinical relevance of individual BMS subjects, which has significant implications for curriculum reform and teaching strategies, an area that is under-researched. Notably, only one prior study has explored this topic in Saudi Arabia; to the best of our knowledge, this is the most comprehensive investigation to date. By incorporating a larger, more diverse sample and detailed subgroup analyses, this study significantly advances current understanding in this domain. Finally, the findings contribute to the ongoing discourse on curriculum reform, offering practical insights for designing educational strategies that enhance student engagement, clinical relevance, and career interest in BMS.

However, the study also had some limitations. Since the study relied on self-reported data, there is a possibility of bias in the responses, as they are based on student perceptions and attitudes, which can be influenced by personal experiences and recent academic exposure. The study’s cross-sectional design also means it can only capture perceptions at a single time point without allowing for the assessment of changes over time or causal relationships. The use of snowball sampling and the online distribution method might have led to potential sampling bias, as less engaged or less tech-savvy students may have been excluded from the survey, limiting the representativeness of the sample. Furthermore, as the study was conducted in colleges in and around Riyadh, the findings may not be fully generalizable to healthcare students in other regions or countries, where educational systems and cultural contexts may differ. Q3 from the attitude questionnaire (‘Psychological factors are just as important as physical ones in the healing process’) was retained from the original validated instrument but represents a broader biopsychosocial perspective that may not directly align with the core construct of attitudes toward basic medical sciences. Findings related to this item are therefore interpreted with caution as exploratory observations. Additionally, a large proportion of respondents reported a GPA of 4–5, which may introduce selection bias, as these students could be more academically motivated or engaged with BMS. This may limit the generalizability of our findings to students with a wider range of academic performance. Lastly, the lack of qualitative questions in the questionnaire limits the depth of understanding regarding students’ perceptions, especially for subjects like biochemistry and microbiology, which were rated as less relevant by some students. Open-ended questions could have provided more detailed insights into the reasons behind these ratings.

### Future directions

Future research should explore longitudinal trends in students’ perceptions of basic medical sciences (BMS), tracking changes from preclinical years into clinical practice to understand their impact on clinical competence and academic performance. Incorporating qualitative methods, such as interviews and focus groups, could reveal deeper motivations, barriers, and career preferences. Expanding studies across multiple institutions and regions would enable cross-cultural comparisons and identification of best practices. Interventional studies testing integrated modules, flipped classrooms, early clinical exposure, and research-linked learning activities are recommended to evaluate strategies for enhancing BMS engagement, particularly in underappreciated disciplines like biochemistry and microbiology. Investigating faculty perspectives on curriculum reform, including barriers to integration, can further guide targeted faculty development. Additionally, structured student feedback mechanisms and the promotion of early research exposure may strengthen the clinical relevance of BMS and support informed career choices. Research into hybrid models blending traditional teaching strengths with integrated approaches could refine curricula to optimize both foundational knowledge and clinical applicability.

## Conclusion

This study highlights the critical role of students’ perceptions of basic medical sciences (BMS) in shaping curriculum preferences and professional attitudes. Students valued BMS for its clinical relevance and strongly supported integrated, longitudinal teaching models and active learning approaches such as problem-based learning. However, despite academic interest, reluctance to pursue BMS-related careers persists, indicating a need for improved mentorship, career guidance, and early research exposure. Significant differences across sex, academic programs, and curriculum types emphasize the need for tailored, inclusive educational strategies. Overall, fostering a clinically integrated, student-centered approach to BMS teaching is essential for enhancing academic engagement, clinical competence, and sustained professional interest. The findings support integrated, PBL-informed curricula; however, students’ continued appreciation for structured lectures and stepwise learning, even within PBL programs, suggests hybrid models blending both approaches may be optimal, with flexibility to accommodate program-specific competency goals. Further studies comparing learning outcomes across curricular models and evaluating program-specific adaptations are warranted to guide evidence-based reforms.

## Data Availability

The original contributions presented in the study are included in the article/Supplementary material, further inquiries can be directed to the corresponding author.

## Footnotes

1
http://www.raosoft.com/samplesize.html

## Supplementary material

SUPPLEMENTARY FIGURE S1 Flow chart illustrating the sample collection and selection as per STROBE guidelines.
