# Supplementary figures and images for: Perceptions, attitudes, and curriculum reflections: exploring healthcare students’ engagement with basic medical sciences in Saudi Arabia
Source: Front Med (Lausanne). 2026 Mar 31;13:1791516. doi: 10.3389/fmed.2026.1791516 (PMC13076158; doi:10.3389/fmed.2026.1791516)

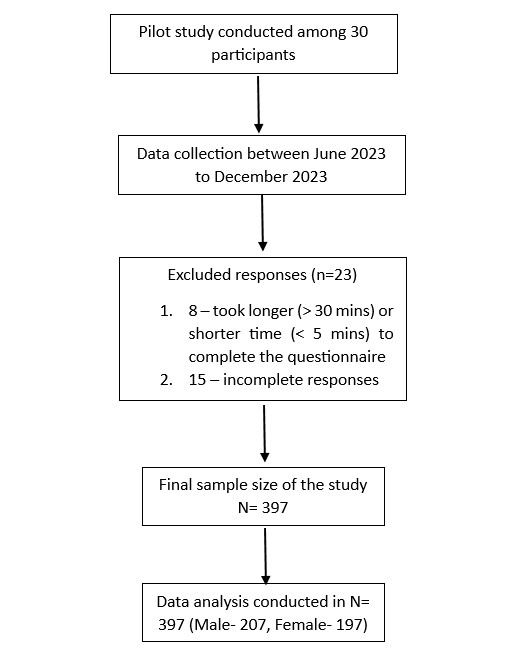

Supplement: SUPPLEMENTARY FIGURE S1 — Flow chart illustrating the sample collection and selection as per STROBE guidelines. [file Image_1.jpeg]
