# Supplementary material for: Perceptions, attitudes, and curriculum reflections: exploring healthcare students’ engagement with basic medical sciences in Saudi Arabia
Source: Front Med (Lausanne). 2026 Mar 31;13:1791516. doi: 10.3389/fmed.2026.1791516 (PMC13076158; doi:10.3389/fmed.2026.1791516)
Supplement: Supplementary file 2 [file Supplementary_file_1.DOCX]

**Supplementary table S1: Factor analysis of the 9-item questionnaire assessing students' perceptions toward Basic Medical Sciences**

| **Rotated Component Matrix^a^** | | |
| --- | --- | --- |
|  | Component | |
|  | 1 | 2 |
| Q1 |  | .813 |
| Q2 |  | .835 |
| Q3 | .524 |  |
| Q4 | .618 |  |
| Q5 | .717 |  |
| Q6 | .662 |  |
| WestQ9 | .695 | -.132 |
| Extraction Method: Principal Component Analysis.  Rotation Method: Varimax with Kaiser Normalization.^a^ | | |
| 1. Rotation converged in 3 iterations. | | |

**Supplementary table S2: Factor analysis of the questionnaire assessing students' perceptions toward Basic Medical Sciences after removal of two items**

| **Rotated Component Matrix^a^** | | |
| --- | --- | --- |
|  | **Component** | |
|  | **1** | **2** |
| **WestQ1** |  | **.793** |
| **WestQ2** |  | **.788** |
| **WestQ3** | **.502** |  |
| **WestQ4** | **.562** |  |
| **WestQ5** | **.707** |  |
| **WestQ6** | **.652** |  |
| **WestQ7** | **.489** | **.345** |
| **WestQ8** | **.323** | **-.274** |
| **WestQ9** | **.687** | **-.122** |
| **Extraction Method: Principal Component Analysis.**  **Rotation Method: Varimax with Kaiser Normalization.^a^** | | |
| **a. Rotation converged in 3 iterations.** | | |

**Supplementary table S3: Factor analysis of the questionnaire assessing students' perceptions of their curriculum**

| **Rotated Component Matrix^a^** | | |
| --- | --- | --- |
|  | Component | |
|  | 1 | 2 |
| CQ1 |  | .839 |
| CQ2 | .518 | -.503 |
| CQ3 |  | .880 |
| CQ4 | .707 |  |
| CQ5 | .810 |  |
| CQ6 | .824 |  |

**Supplementary table S4: Clinical Relevance Rating** **of the Basic Medical Science Disciplines**

| Basic medical science | Irrelevant | | Moderately relevant | | Relevant | |
| --- | --- | --- | --- | --- | --- | --- |
|  | Frequency (n=397) | Percentage | Frequency (n=397) | Percentage | Frequency (n=397) | Percentage |
| Physiology | 26 | 6.5 | 101 | 25.4 | 270 | 68.0 |
| Anatomy | 16 | 4.0 | 60 | 15.1 | 321 | 80.9 |
| Biochemistry | 129 | 32.5 | 158 | 39.8 | 110 | 27.7 |
| Pathology | 26 | 6.5 | 57 | 14.4 | 314 | 79.1 |
| Pharmacology | 72 | 18.1 | 111 | 28.0 | 214 | 53.9 |
| Microbiology and immunology | 79 | 19.9 | 125 | 31.5 | 193 | 48.6 |

**Supplementary table S5: Collective students' response about perception and attitude towards BMS**

| Question | 50^th^ percentile (Median) | Highly disagree | | Disagree | | Neutral | | Agree | | Highly agree | |
| --- | --- | --- | --- | --- | --- | --- | --- | --- | --- | --- | --- |
|  |  | N | % | N | % | N | % | N | % | N | % |
| A healthcare professional can effectively deal with patients without knowing the details of biological processes involved? | 2.00 | 113 | 28.5 | 106 | 26.7 | 108 | 27.2 | 45 | 11.3 | 25 | 6.3 |
| Most basic science research is far away from clinical practice as its relevance is very minimal in routine practice? | 3.00 | 76 | 19.1 | 88 | 22.2 | 151 | 38.0 | 48 | 12.1 | 34 | 8.6 |
| Psychological factors are just as important as physical factors in the healing process? | 5.00 | 14 | 3.5 | 12 | 3.0 | 56 | 14.1 | 84 | 21.2 | 231 | 58.2 |
| Of all the facets of a good healthcare professional, his/ her knowledge of biological mechanism is the most important one? | 3.00 | 17 | 4.3 | 46 | 11.6 | 155 | 39.0 | 93 | 23.4 | 86 | 21.7 |
| Applying the basic science of medicine to clinical practice is a skill which should be reinforced early on in medical education? | 4.00 | 7 | 1.8 | 15 | 3.8 | 94 | 23.7 | 103 | 25.9 | 178 | 44.8 |
| It is necessary to first learn as many facts as possible in basic medical sciences and then learn to apply them later on in the clinical years ? | 4.00 | 15 | 3.8 | 30 | 7.6 | 110 | 27.7 | 100 | 25.2 | 142 | 35.8 |
| What students should learn in basic medical sciences are the general concepts, so that they might have a good working knowledge without having to know all the facts? | 4.00 | 21 | 5.3 | 49 | 12.3 | 119 | 30.0 | 102 | 25.7 | 106 | 26.7 |
| Staff members excite students’ curiosity by teaching basic medical sciences? | 3.00 | 59 | 14.9 | 77 | 19.4 | 114 | 28.7 | 69 | 17.4 | 78 | 19.6 |
| The information and experiences I have received from basic medical science courses are fundamental to my future role as a healthcare professional? | 4.00 | 13 | 3.3 | 26 | 6.5 | 92 | 23.2 | 133 | 33.5 | 133 | 33.5 |

**Supplementary table S6: Collective students' response about perception and attitude towards curriculum**

| Question | 50^th^ percentile (Median) | Highly disagree | | Disagree | | Neutral | | Agree | | Highly agree | |
| --- | --- | --- | --- | --- | --- | --- | --- | --- | --- | --- | --- |
|  |  | N | % | N | % | N | % | N | % | N | % |
| Conventional teaching of basic science courses is essential for better understanding of clinical sciences | 3.00 | 63 | 15.9 | 68 | 17.1 | 118 | 29.7 | 90 | 22.7 | 58 | 14.6 |
| Do you feel that problem based teaching makes the relevance of Basic Medical Science Courses in healthcare curriculum more important - | 4.00 | 13 | 3.3 | 34 | 8.6 | 98 | 24.7 | 109 | 27.5 | 143 | 36.0 |
| Do you feel that the Conventional lectures and practical sessions are the best way to understand Basic Medical Science Courses | 3.00 | 76 | 19.1 | 95 | 23.9 | 111 | 28.0 | 71 | 17.9 | 44 | 11.1 |
| Do you feel that the Basic Medical Science Courses should be spread throughout the curriculum (from 1st to final year) to reduce the burden of knowledge overload during the earlier years | 4.00 | 36 | 9.1 | 37 | 9.3 | 80 | 20.2 | 78 | 19.6 | 166 | 41.8 |
| Do you feel that integrating Basic Medical Science Courses with clinical courses would benefit you to be a better healthcare professional | 4.00 | 21 | 5.3 | 17 | 4.3 | 80 | 20.2 | 93 | 23.4 | 186 | 46.9 |
| Do you feel that integrating Basic Medical Science Courses with clinical courses would benefit you to be a better researcher | 5.00 | 18 | 4.5 | 18 | 4.5 | 72 | 18.1 | 90 | 22.7 | 199 | 50.1 |

**Supplementary table S7: Subgroup comparison of the students’ response about perception and attitude towards BMS (non-significant results)**

| Question | Age | | | | | Year of study | | | | | | |
| --- | --- | --- | --- | --- | --- | --- | --- | --- | --- | --- | --- | --- |
|  | 18-20  mean rank (N=122) | 21-23  mean rank (N=191) | 24-27  mean rank (N=76) | 27-30  mean rank (N=8) | P value | 1^st^ year  Mean rank  (N=86) | 2^nd^ year  Mean rank  (N=86) | 3^rd^ year  Mean rank  (N=72) | 4^th^ year  Mean rank  (N=59) | 5^th^ year  Mean rank  (N=45) | Internship  Mean rank  (N=49) | P value |
| Q1 | 202.44 | 195.18 | 205.99 | 171.31 | .723 | 208.48 | 196.73 | 188.42 | 190.08 | 198.81 | 212.81 | .723 |
| Q2 | 196.07 | 199.90 | 200.28 | 210.06 | .977 | 209.84 | 187.94 | 191.13 | 196.26 | 200.30 | 213.05 | .685 |
| Q3 | 204.87 | 200.52 | 189.31 | 165.31 | .364 | 209.58 | 196.63 | 187.63 | 204.03 | 190.10 | 203.42 | .567 |
| Q4 | 187.58 | 201.39 | 215.13 | 162.75 | .236 | 186.15 | 187.42 | 187.78 | 209.29 | 216.67 | 229.76 | .104 |
| Q5 | 202.95 | 196.42 | 196.26 | 226.31 | .761 | 188.22 | 209.19 | 185.26 | 202.22 | 197.68 | 217.55 | .305 |
| Q6 | 208.11 | 191.42 | 204.24 | 191.13 | .494 | 208.80 | 187.92 | 189.96 | 191.77 | 210.94 | 212.28 | .502 |
| Q7 | 188.93 | 203.55 | 207.01 | 167.69 | .444 | 200.17 | 195.48 | 190.15 | 204.26 | 211.30 | 198.51 | .923 |
| Q8 | 197.53 | 206.87 | 181.86 | 196.25 | .398 | 207.30 | 203.07 | 175.93 | 197.48 | 215.51 | 197.85 | .417 |
| Q9 | 200.30 | 198.01 | 195.74 | 233.94 | .749 | 192.56 | 208.45 | 189.19 | 199.08 | 201.53 | 205.69 | .808 |

**Supplementary table S8: Subgroup comparison of the students’ response about perception and attitude towards curriculum**

| Question | Age | | | | | Year of study | | | | | | |
| --- | --- | --- | --- | --- | --- | --- | --- | --- | --- | --- | --- | --- |
|  | 18-20  mean rank (N=122) | 21-23  mean rank (N=191) | 24-27  mean rank (N=76) | 27-30  mean rank (N=8) | P value | 1^st^ year  Mean rank  (N=86) | 2^nd^ year  Mean rank  (N=86) | 3^rd^ year  Mean rank  (N=72) | 4^th^ year  Mean rank  (N=59) | 5^th^ year  Mean rank  (N=45) | Internship  Mean rank  (N=49) | P value |
| Q1 | 191.18 | 209.10 | 188.38 | 178.13 | .342 | 210.08 | 175.40 | 195.06 | 226.47 | 202.20 | 190.73 | .099 |
| Q2 | 203.77 | 198.48 | 192.88 | 196.81 | .897 | 200.34 | 199.99 | 214.42 | 181.58 | 179.22 | 211.40 | .278 |
| Q3 | 187.30 | 203.43 | 206.66 | 198.88 | .539 | 199.19 | 184.76 | 193.36 | 210.34 | 220.18 | 198.86 | .531 |
| Q4 | 213.13 | 187.88 | 204.63 | 195.63 | .166 | 216.84 | 203.76 | 196.44 | 192.59 | 184.80 | 183.85 | .381 |
| Q5 | 210.50 | 197.95 | 182.49 | 205.75 | .220 | 208.68 | 209.37 | 209.03 | 192.19 | 174.34 | 179.92 | .129 |
| Q6 | 211.43 | 196.03 | 183.90 | 223.69 | .148 | 214.18 | 201.17 | 200.87 | 189.38 | 214.18 | 201.17 | .444 |

**Supplementary table S9: Regression analysis of the determinants of students’ positive and negative attitude towards BMS**

| Determinant | Positive attitude towards BMS | | | Negative attitude towards BMS | | |
| --- | --- | --- | --- | --- | --- | --- |
|  | B value | 95% confidence interval | P value | B value | 95% confidence interval | P value |
| Gender  Male  Female (Ref) | -0.51 | -0.133 to 0.032 | 0.23 | 0.134 | 0.009 to 0.260 | **0.036** |
|  | - | - | - | - | - | - |
| Type of curriculum  Conventional | 0.135 | -0.019 to 0.289 | 0.087 | -0.039 | -0.274 to 0.196 | 0.744 |
| PBL | 0.220 | 0.060 to 0.380 | **0.007** | -0.070 | -0.313 to 0.173 | 0.570 |
| Others (Ref) | - | - | - | - | - | - |
| Academic programme  Medicine | -0.122 | -0.293 to 0.050 | 0.164 | 0.296 | 0.036 to 0.557 | **0.026** |
| Dentistry | 0.038 | -0.126 to 0.203 | 0.646 | 0.199 | -0.051 to 0.448 | 0.119 |
| Allied Health Science | -0.073 | -0.270 to 0.124 | 0.466 | 0.308 | 0.008 to 0.607 | **0.044** |
| Nursing | -0.014 | -0.205 to 0.205 | 0.885 | 0.172 | -0.118 to 0.462 | 0.245 |
| Others (Ref) | - | - | - | - | - | - |
| Type of university  Public | 0.016 | -0.115 to 0.146 | 0.816 | -0.224 | -0.423 to -0.026 | **0.027** |
| Private (Ref) | - | - | - | - | - | - |
| Year of study  1^st^ year | -0.078 | -0.224 to 0.068 | 0.295 | -0.062 | -0.283 to 0.160 | .585 |
| 2^nd^ year | -0.091 | -0.237 to 0.054 | 0.220 | -0.201 | -0.423 to 0.020 | .075 |
| 3^rd^ year | -0.151 | -0.302 to -0.001 | **0.048** | -0.216 | -0.445 to 0.012 | .063 |
| 4^th^ year | -0.081 | -0.236 to 0.073 | 0.303 | -0.145 | -0.380 to 0.091 | .228 |
| 5^th^ year | -0.099 | -0.267 to 0.068 | 0.244 | -0.106 | -0.360 to 0.148 | .414 |
| Internship (Ref) | - | - | - | - | - | - |

**Supplementary table S10: Regression analysis of the determinants of students’ attitude towards conventional and integrated curriculum**

| Determinant | Attitude towards conventional curriculum | | | Attitude towards integrated curriculum | | |
| --- | --- | --- | --- | --- | --- | --- |
|  | B value | 95% confidence interval | P value | B value | 95% confidence interval | P value |
| Gender  Male  Female (Ref) | -0.061 | -0.199 to 0.076 | 0.382 | -0.015 | -0.111 to 0.081 | 0.760 |
|  | - | - | - | - | - | - |
| Type of curriculum  Conventional | 0.158 | -0.098 to 0.415 | 0.226 | -0.033 | -0.212 to 0.146 | 0.721 |
| PBL | -0.479 | -0.744 to -0.214 | **<0.001** | 0.285 | 0.100 to 0.470 | **0.003** |
| Others (Ref) | - | - | - | - | - | - |
| Academic programme  Medicine | 0.159 | -0.125 to 0.444 | 0.273 | -0.138 | -0.336 to 0.061 | 0.175 |
| Dentistry | 0.012 | -0.261 to 0.284 | 0.934 | 0.039 | -0.151 to 0.230 | 0.687 |
| Allied Health Science | -0.121 | -0.448 to 0.206 | 0.469 | 0.006 | -0.222 to 0.234 | 0.959 |
| Nursing | 0.005 | -0.312 to 0.321 | 0.977 | 0.073 | -0.148 to 0.294 | 0.517 |
| Others (Ref) | - | - | - | - | - | - |
| Type of university  Public | -0.213 | -0.430 to 0.004 | 0.054 | 0.039 | -0.112 to 0.191 | 0.610 |
| Private (Ref) | - | - | - | - | - | - |
| Year of study  1^st^ year | 0.038 | -0.204 to 0.280 | 0.758 | 0.160 | -0.009 to 0.330 | 0.063 |
| 2^nd^ year | -0.074 | -0.316 to 0.167 | 0.546 | 0.075 | -0.093 to 0.244 | 0.381 |
| 3^rd^ year | -0.009 | -0.259 to 0.240 | 0.941 | 0.108 | -0.066 to 0.282 | 0.224 |
| 4^th^ year | .0154 | -0.103 to 0.411 | 0.241 | -0.004 | -0.183 to 0.176 | 0.967 |
| 5^th^ year | 0.141 | -0.137 to 0.419 | 0.321 | -0.092 | -0.286 to 0.102 | 0.353 |
| Internship (Ref) | - | - | - | - | - | - |
