# Supplementary material for: Perceptions, attitudes, and curriculum reflections: exploring healthcare students’ engagement with basic medical sciences in Saudi Arabia
Source: Front Med (Lausanne). 2026 Mar 31;13:1791516. doi: 10.3389/fmed.2026.1791516 (PMC13076158; doi:10.3389/fmed.2026.1791516)
Supplement: Supplementary file 3 [file Data_Sheet_1.PDF]

# Questionnaire used

Perception and interest about BMS (adapted from Daniel T et al., (2021)

1. Are you interested in BMS courses – Verymuch, Minimal, Null
2. Do you plan a career in BMS- Ofcourse, maybe, never
3. Will you guide your junior to join a career in BMS - Ofcourse, maybe, never
4. What is the reason to not join in BMS as a career
    a. Not interested
    b. Family pressure
    c. Less financial growth
    d. Less chance of promotion
    e. Less thrilling field
    f. No role model
    g. None of the above
5. Should your BMS teachers encourage you to join this field- Yes/ No
6. Should be number of teaching hours increase in BMS courses- Yes/ No
7. Will an integrated curriculum increase your interest to BMS- Yes/ No/ I don't know
8. Will preclinical/ animal research opportunities in BMS interest you - Yes/ No/ I don't know
9. Which of the following courses in BMS has clinical relevance (Irrelevant/ moderately relevant/ relevant)
    a. Anatomy
    b. Physiology
    c. Biochemistry
    d. Pathology
    e. Pharmacology
    f. Microbiology and immunology

Attitudes of students towards BMS (Adapted from M West et al.

1982) (Highly disagree/ Disgree/ Neutral/ Agree/ Highly Agree)

1. An healthcare professional can effectively deal with patients without knowing the details of biological processes involved
2. Most basic science research is far away from clinical practice as its relevance is very minimal in routine practice
3. Psychological factors are just as important as physical factors in the healing process
4. Of all the facets of a good healthcare professional, his/ her knowledge of biological mechanism is most important
5. Applying the basic science of medicine to clinical practice is a skill which should be reinforced early on in medical education
6. It is first necessary to learn as many facts as possible in the basic sciences and then learn to apply them later on in the clinical year
7. What students should learn in basic sciences are the general concepts, in order that they might have a good working knowledge without having to know all the facts.

8. Staff members excite students' curiosity through the teaching of the basic sciences.
9. The information and experiences I have received to date are fundamental to my future role as a healthcare professional

About curriculum

1. Which type of curriculum is followed for BMS courses in your program? Conventional course based/ PBL
2. Conventional teaching of basic science courses is essential for better understanding of clinical sciences - (Highly disagree/ Disagree/ Neutral/ Agree/ Highly Agree)
3. Problem based teaching makes the relevance of BMS courses in an healthcare curriculum more important- (Highly disagree/ Disagree/ Neutral/ Agree/ Highly Agree)
4. Conventional lectures and practical sessions is the best way to understand BMS courses (Highly disagree/ Disagree/ Neutral/ Agree/ Highly Agree)
5. BMS course should be spread throughout the curriculum (from 1st to final year) to reduce the burden of knowledge overload during the earlier years (Highly disagree/ Disagree/ Neutral/ Agree/ Highly Agree)
6. Integrating BMS courses with clinical course would benefit me to be a better healthcare professional (Highly disagree/ Disagree/ Neutral/ Agree/ Highly Agree)
7. Integrating BMS courses with clinical course would benefit me to be a better researcher (Highly disagree/ Disagree/ Neutral/ Agree/ Highly Agree)
